# Design, fabrication and characterization of mesoporous yolk–shell nanocomposites as a sustainable heterogeneous nanocatalyst for synthesis of ortho-aminocarbonitrile tetrahydronaphthalenes

**DOI:** 10.1038/s41598-023-50021-7

**Published:** 2023-12-18

**Authors:** Somayeh Kazempour, Hossein Naeimi

**Affiliations:** Department of Organic Chemistry, Faculty of Chemistry, University of Kashan, Kashan, 87317-51167 Islamic Republic of Iran

**Keywords:** Chemistry, Materials science, Nanoscience and technology

## Abstract

A new structure of mesoporous spherical nanocomposites was designed and easily prepared from the reaction between NiCuFe_2_O_4_ nanoparticles and mesoporous silica in three steps. The prepared multi-yolk@shell NiCuFe_2_O_4_@mSiO_2_ mesoporous sphere was characterized by using FT-IR, XRD, VSM, EDX, BET, FE-SEM and HR-TEM techniques. This unique mesoporous nanocomposite sphere as a heterogeneous nanocatalyst has demonstrated highly catalytic activity for the green synthesis of tetrahydronaphthalene derivatives in 92–98% yields at reaction times of 60–75 min. This process was carried out through multi-component reaction of the cyclic ketone, malononitrile and aromatic aldehyde under solvent-free conditions. Furthermore, the procedure was optimized on the basis of catalyst loading amounts, various solvents and temperature conditions. This novel methodology exposes obvious benefits such as; catalyst reusability, easy reaction procedure, simplicity of work-up, excellent product yields and short reaction times.

## Introduction

Recently, nanotechnology and nanoscale materials, especially magnetic nanoparticles (MNPs) have attracted a lot of attention and shown many applications in research and industrial fields in the chemical, medical and environmental sectors. For example, the application of magnetic nanoparticles in removal of pollutants or reducing toxicity is promising^1^. The properties of MNPs can be related to the physical characteristics of nanoparticles such as; shape, chemical composition, size, crystal structure morphology and surface area effects. These MNPs are usually very different from the properties of bulk materials^2^ and coated with a shell to show the core–shell nanoparticles (CSNPs)^3–5^. The core–shell nanoparticles are often created by the formation of a dense layer surrounding center. The properties of CSNPs are mostly determined by the type of core and the contained shell, as well as the sizes of both core and shell and the combination of both. Although CSNPs offer several improved features and advantages over traditional MNPs such as; narrow size distribution, higher chemical stability and magnetic moment can be tuned with the nanoparticle cluster size^6^. However, these particles still have some limitations such as; a dense shell coating that restricts the native surface of MNPs and the accessibility of reagents to the catalytic active sites of MNPs^7^. Therefore, to remove these limitations, a type of core–shell nanoparticles was considered.

Yolk–shell nanoparticles (YSNPs) are a novel class of unique core–shell nanoparticles that are commonly referred to as hollow shells with a void area between the solid particle core and shell, allowing the core to move freely within the shell^8,9^. Because of their unique optical, chemical, mechanical, and electrical properties, the YSNPs have recently received a number of attention from researchers, and they have a lot of potential in catalysis^10–12^, lithium-ion batteries^13–15^, biosensors^16,17^, bioimaging^18–20^, surface-enhanced Raman scattering^21–23^, and biomedicine applications^24–26^.

Multicomponent reactions (MCRs) are one-pot reactions using more than two substrate materials, where most of the atoms of the starting materials are incorporated in the final product. These reactions are economical and environmentally friendly methods for organic synthesis^27,28^.

One of the multicomponent reactions is the synthesis of tetrahydronaphtalenes. Tetralin or tetrahydronaphthalene is obtained from the hydrogenation of naphthalene^29^. This compound is a colorless liquid with a chemical formula, C_10_H_12_ that is used as a hydrogenation solvent^30^. Its laboratory synthesis was first prepared by Darnz. The ortho-aminocarbonitriles with two chiral centers that exhibit optical activity are also crucial intermediates in the synthesis of a number of heterocyclic compounds. Several interesting organic chemistry works related to the ortho-aminocarbonitrile tetrahydronaphthalene derivatives has led to the development of numerous techniques in recent years^31^.

Recently years, the different catalysts are used for synthesis of tetralin compound such as; Fe_3_O_4_@SiO_2_@mSiO_2_-Pd^32^, Pd/S-1@ZSM-5 core–shell^33^, CaMg@ mesoporous silica yolk shell^34^, CaMgFe_2_O_4_ magnetic nanocatalyst^35^ and NaOH catalyst^36^. However, these catalysts have disadvantages such as; long reaction times, low reaction yields and high loading catalyst. While, in this work, by using the prepared nanocatalyst, the tetrahydronaphthalene derivatives are easily produced in excellent yields, low catalyst amounts and low reaction times.

In continuation of ongoing our team work on the basis of the catalytic reactions^37,38^, the purpose of this research was to create a new catalyst with high activity through simple procedure. Therefore, the multi-yolk@shell NiCuFe_2_O_4_@mSiO_2_ sphere was fabricated and used for multi-component reaction of cyclic ketone, malononitrile and aromatic aldehyde under solvent-free conditions. In this methodology, the tetrahydronaphthalenes were purely obtained in highly efficiency and short reaction times.

## Experimental

### Materials and apparatus

All chemicals and reagents, namely calcium nitrate trihydrate (99%), nickel nitrate hexahydrate (99%), iron nitrate nonanehydrate (99%), tetraethyl orthosilicate, glucose, barbituric acid, 1-naphthylamine and the respective benzaldehyde derivatives, were purchased from Merck Chemical Company. ^1^H NMR spectra were taken with 400 MHz NMR spectrometer for solution NMR analysis. FT-IR spectra were obtained with Shimadzu’s instrument. Electrothermal programmable digital melting point apparatus was used to measure the melting points. BGMN/Profex/AutoQuan software reported the results of the X-ray diffraction studies. The nanoparticles were examined using a field emission scanning electron microscope (FE-SEM), on the Zeiss that run at an accelerating voltage of 15 kV. The magnetic properties of nanostructure was measured by a vibrating sample magnetometer (VSM) with PPMS-9T at 300 K. The Brunauer–Emmett–Teller (BET) specific area and pore volume was measured via the nitrogen adsorption–desorption isotherms method using BELSORP MINI II. The high resolution transmission electron microscopy (HR-TEM) was prepared by a FEI TECNAI F20 at 200 kV instrument.

### General procedure for preparation of NiCuFe_2_O_4_@mSiO_2_

In accordance to the previously reported procedure^39^, in the first step, to a glucose (4.95 mg) dissolved in 25 mL of distilled water, the Fe(NO_3_)_3_.9H_2_O (0.8 mg), Ni(NO_3_)_2_⋅6H_2_O (0.14 mg), and Cu(NO_3_)_2_⋅3H_2_O (0.12 mg) salts were dispersed in 10 mL distilled water. After being stirred for about 30 min, the mixture was transferred into a Teflon-lined stainless steel autoclave. The autoclave was maintained at 180 °C for 24 h and then naturally cooled to ambient temperature. The precipitate was filtered, washed with water and ethanol, then dried at 100 °C and NiCuFe_2_O_4_@C was obtained. In the second step, NiCuFe_2_O_4_@C (0.1 g), CTAB (0.15 g), and NH_3_⋅H_2_O (580 µL) were dispersed into the mixture of 40 mL H_2_O and ethanol on the magnetic stirring for 30 min. Then, 150 µL TEOS added to mixture reaction and the reaction maintained for 6 h at room temperature. The black solid was obtained, washed with water and ethanol; then dried in the oven at 70 °C and then, calcination for 3 h under air atmosphere at 600 °C. The final, multi yolk@shell NiCuFe_2_O_4_@mSiO_2_ sphere was made.

### A general procedure for the tetrahydronaphthalene derivatives

In this section, cyclohexanon derivatives (1 mmol), malononitrile (2 mmol), aromatic aldehyde (1 mmol), and 1.0 mg NiCuFe_2_O_4_@mSiO_2_ as a catalyst are mixed under stirring at 50 °C. The progress of the reaction was monitored by thin-layer chromatography (TLC). After completion of the reaction, the ethanol (3 mL) was added and the catalyst was separated by an external magnet. The reaction mixture filtered and washed with ethanol (3 mL), then the pure products are obtained.

#### 2-Amino-4-phenyl-4a,5,6,7-tetrahydronaphthalene-1,3,3(4*H*)-tricarbonitrile (4a)

White solid; m.p.: 255–257 °C (Lit.^40^ m.p 255–258 °C); IR (KBr) ʋ = 3419, 3339, 2933, 2856, 2210, 1648, 1451, 1276, 1037, 784, 580 cm^−1^; ^1^H NMR (400 MHz, DMSO-d_6_): δ = 7.37–7.60 (m, 7H, NH_2_, CH), 5.71 (s, 1H), 3.52 (d, *J* = 12.0 Hz, 1H), 2.76–2.83 (m, 1H), 2.00–2.20 (m, 2H), 1.64 (t, *J* = 12.0 Hz, 1H), 1.39–1.48 (m, 2H), 0.79–0.89 (m, 1H).

#### 2-Amino-4-(4-bromophenyl)-4a,5,6,7-tetrahydronaphthalene-1,3,3(4*H*)-tricarbonitrile (4b)

White solid; m.p.: 244–246 °C (Lit.^40^ m.p 243–245 °C); IR (KBr) ʋ = 3428, 3346, 2946, 2210, 1642, 1522, 1348, 1276, 1113, 807 cm^−1^; ^1^H NMR (400 MHz, DMSO-d_6_): δ = 8.32 (t, *J* = 8.0 Hz, 2H), 7.91 (d, *J* = 8.0 Hz, 1H), 7.73 (d, *J* = 8.0 Hz, 1H), 7.43 (s, 2H, NH_2_), 5.74 (s, 1H), 3.87 (d, *J* = 12.0 Hz, 1H), 2.84–2.91 (m, 1H), 2.02–2.20 (m, 2H), 1.67 (d, *J* = 12.0 Hz, 1H), 1.39–1.48 (m, 2H), 0.81–0.90 (m, 1H).

#### 2-Amino-4-(4-nitrophenyl)-4a,5,6,7-tetrahydronaphthalene-1,3,3(4*H*)-tricarbonitrile (4c)

Yellow solid; m.p.: 264–266 °C (Lit.^40^ m.p 263–266 °C); IR (KBr) ʋ = 3419, 3341, 2947, 2856, 2215, 1646, 1604, 1455, 1256, 1025, 833, 570 cm^−1^, ^1^H NMR (400 MHz, DMSO-d_6_): δ = 8.33 (t, *J* = 12.0 Hz, 2H), 7.91 (d, *J* = 8.0 Hz, 1H), 7.72 (d, *J* = 8.0 Hz, 1H), 7.43 (s, 2H, NH_2_), 5.74 (s, 1H), 3.90 (d, *J* = 12.0 Hz, 1H), 2.84–2.91 (m, 1H), 2.00–2.21 (m, 2H), 1.66–1.70 (m, 1H), 1.39–1.48 (m, 2H), 0.81–0.90 (m, 1H).

#### 2-Amino-4-(4-(dimethylamino)phenyl)-4a,5,6,7-tetrahydronaphthalene-1,3,3(4*H*)-tricarbo-nitrile (4d)

Yellow solid; m.p.: 263–265 °C (Lit.^40^ m.p 264–265 °C); IR (KBr) ʋ = 3427, 3341, 2921, 2203, 1614, 1518, 1379, 1184, 814 cm^−1^, ^1^H NMR (400 MHz, DMSO-d_6_): δ = 7.17–7.34 (m, 4H, NH_2_, CH), 6.60–6.80 (m, 2H), 5.08 (s, 1H), 3.31 (d, *J* = 12.0 Hz, 1H), 2.92 (s, 6H, OCH_3_), 2.66–2.75 (m, 1H), 2.49 (s, H), 2.00–2.19 (m, H), 1.64–1.70 (m, 1H), 1.41–1.53 (m, 2H), 0.77–0.86 (m, 1H).

#### 2-Amino-4-(4-fluorophenyl)-4a, 5,6,7-tetrahydronaphthalene-1,3,3(4*H*)-tricarbonitrile (4e)

White solid; m.p.: 262–264 °C (Lit.^40^ m.p 263–267 °C); IR (KBr) ʋ = 3418, 3378, 2951, 2869, 2211, 1621, 1511, 1449, 1390, 1231, 1163, 844 cm^−1^, ^1^H NMR (400 MHz, DMSO-d_6_): δ = 7.63 (s, H), 7.48 (s, H), 7.21–7.37 (m, 4H, NH_2_, CH), 5.71 (s, 1H), 3.62 (d, *J* = 12.0 Hz, 1H), 2.75–2.82 (m, 1H), 2.05–2.20 (m, 2H), 1.66–1.76 (m, 1H), 1.40–1.45 (m, 2H), 0.82–0.88 (m, 1H) ppm.

#### 2-Amino-4-(4-chlorophenyl)-4a,5,6,7-tetrahydronaphthalene-1,3,3(4*H*)-tricarbonitrile (4f)

White solid; m.p.: 248–250 °C (Lit.^40^ m.p 246–249 °C); IR (KBr) ʋ = 3443, 3339, 3033, 2923, 2225, 1643, 1583, 1289, 1093, 1007, 825, 748, 501 cm^−1^, ^1^H NMR (400 MHz, DMSO-d_6_): δ = 7.46–7.61 (m, 4H), 7.38 (s, 2H, NH_2_), 5.71 (s, 1H), 3.63 (d, *J* = 12.0 Hz, 1H), 2.75–2.83 (m, 1H), 2.01–2.20 (m, 2H), 1.65–1.69 (m, 1H), 1.42–1.46 (m, 2H), 0.79–0.89 (m, 1H) ppm.

#### 2-Amino-4-(4-methoxyphenyl)-4a,5,6,7-tetrahydronaphthalene-1,3,3(4*H*)-tricarbonitrile (4g)

White solid; m.p.: 261–262 °C (Lit.^40^ m.p 258–261 °C); IR (KBr) ʋ = 3421, 3334, 2953, 2213, 1649, 1600, 1451, 1390, 1160, 1037, 706, 594 cm^−1^, ^1^H NMR (400 MHz, DMSO-d_6_): δ = 6.95–750 (m, 6H, NH_2_, CH), 5.70 (s, 1H), 3.78 (s, 3H, OCH_3_), 3.46 (d, *J* = 12.0 Hz, 1H), 2.70–2.78 (m, 1H), 2.00–2.19 (m, 2H), 1.66–1.70 (m, 1H), 1.43–1.49 (m, 2H), 0.77–0.87 (m, 1H) ppm.

#### 2-Amino-4-(4-methylphenyl)-4a,5,6,7-tetrahydronaphthalene-1,3,3(4*H*)-tricarbonitrile (4h)

White solid; m.p.: 235–237 °C (Lit.^41^ m.p 234–236 °C); IR (KBr) ʋ = 3437, 3341, 2956, 2210, 1645, 1599, 1425, 1264, 1044, 818, 513 cm^−1^, ^1^H NMR (400 MHz, DMSO-d6): δ = 7.17–7.47 (m, 6H, NH_2_, CH), 5.70 (s, 1H), 3.47 (d, *J* = 12.0 Hz, 1H), 2.73–2.79 (m, 1H), 2.49 (s, 3H, CH_3_), 2.02–2.19 (m, 2H), 1.64–1.68 (m, 1H), 1.41–1.49 (m, 2H), 0.77–0.87 (m, 1H) ppm.

#### 2-Amino-4-(3-bromophenyl)-4a,5,6,7-tetrahydronaphthalene-1,3,3(4*H*)-tricarbonitrile (4i)

White solid; m.p.: 250–253 °C (Lit.^40^ m.p 248–253 °C); IR (KBr) ʋ = 3417, 3339, 2940, 2859, 2210, 1650, 1568, 1476, 1339, 1212, 1076, 887, 796, 688, 578 cm^−1^, ^1^H NMR (400 MHz, DMSO-d_6_): δ = 7.39–7.77 (m, 6H, NH_2_, CH), 5.72 (s, 1H), 3.64 (d, *J* = 12.0 Hz, 1H), 2.78–2.86 (m, 1H), 2.00–2.20 (m, 2H), 1.66–1.72 (m, 1H), 1.43–1.53 (m, 2H), 0.82–0.92 (m, 1H) ppm.

#### 2-Amino-5-methyl-4-phenyl-4a,5,6,7-tetrahydronaphthalene-1,3,3(4*H*)-tricarbonitrile (4j)

White solid; m.p.: 260–262 °C; IR (KBr) ʋ = 3417, 3357, 2940, 2859, 2210, 1650, 1568, 1476, 1393, 1278, 1076, 578 cm^−1^, ^1^H NMR (400 MHz, DMSO-d6): δ = 7.37–7.60 (m, 7H, NH_2_, CH), 5.71(s, 1H), 3.53 (d, *J* = 8.0 Hz, 1H), 2.82–2.88 (m, 1H), 1.65–1.74 (m, H), 1.30–1.40 (m, 1H), 0.89–0.94 (m, H), 0.81 (d, *J* = 8.0 Hz, 3H, CH_3_), 0.60–0.66 (m, 1H) ppm.

#### 2-Amino-5-methyl-4-(4-bromophenyl)-4a,5,6,7-tetrahydronaphthalene-1,3,3(4*H*)-tricarbo-nitrile (4k)

White solid; m.p.: 253–257 °C; IR (KBr) ʋ = 3442, 3345, 2946, 2859, 2209, 1644, 1600, 1488, 1391, 1268, 1074, 1009, 563 cm^−1^, ^1^H NMR (400 MHz, DMSO-d_6_): δ = 7.58–7.67 (m, 2H), 7.45–748 (m, 1H), 7.16–7.28 (m, 1H), 6.05(s, 1H), 4.84 (s, 2H, NH_2_), 3.07 (d, *J* = 12.0 Hz, 1H), 2.87–2.97 (m, 1H), 2.33–2.40 (m, 1H), 1.71–1.81 (m, 2H), 1.52–1.60 (m, 1H), 0.92 (d, *J* = 8.0 Hz, 3H), 0.68–0.75 (m, 1H) ppm.

#### 2-Amino-5-methyl-4-(4-hydroxyphenyl)-4a,5,6,7-tetrahydronaphthalene-1,3,3(4*H*)-tricarbo nitrile (4l)

White solid; m.p.: 256–257 °C; IR (KBr) ʋ = 3443, 3344, 2947, 2866, 2209, 1646, 1518, 1449, 1384, 1271, 1170, 831, 575 cm^−1^, ^1^H NMR (400 MHz, DMSO-d_6_): δ = 9.66 (s, H, OH), 7.17–7.39 (m, 4H, NH_2_, CH), 6.76–6.87 (m, 2H), 5.68 (s, 1H), 3.39 (s, 1H), 2.76–2.83 (m, 1H), 2.21–2.28 (m, 1H), 1.67 (d, *J* = 12.0 Hz, 2H), 1.38–1.45 (m, 1H), 0.81 (d, *J* = 2.0 Hz, 3H), 0.59–0.64 (m, 1H) ppm.

#### 2-Amino-5-methyl-4-(4-chlorophenyl)-4a,5,6,7-tetrahydronaphthalene-1,3,3(4*H*)-tricarbo-nitrile (4m)

White solid; m.p.: 236–240 °C; IR (KBr) ʋ = 3423, 3345, 2947, 2856, 2211, 1643, 1493, 1452, 1391, 1268, 1093, 825, 511 cm^-1^; ^1^H NMR (400 MHz, DMSO-d_6_): δ = 7.27–7.53 (m, 4H), 6.06 (s, 1H), 4.93 (s, 2H, NH_2_), 2.90–2.96 (m, 1H), 2.33–2.41 (m, 1H), 2.19 (s, 1H), 1.73–1.80 (m, 1H), 1.53–1.61 (m, 1H), 0.91–0.93 (d, *J* = 8.0 Hz, 3H), 0.72–0.79 (m, 1H) ppm.

## Results and discussions

### Preparation and characterization of catalyst

**Figure 1:**
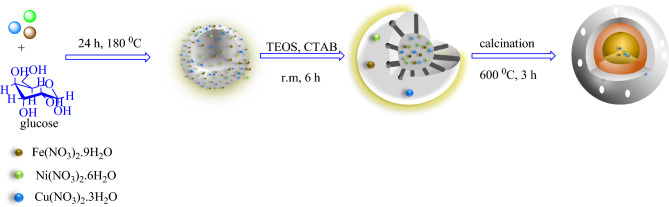
Preparation of multi yolk@shell NiCuFe_2_O_4_@mSiO_2_ spheres.

The multi-yolk@shell NiCuFe_2_O_4_@mSiO_2_ has been designed and easily prepared in a triple step procedure in accordance to the literature^42^ that completely described in “General procedure for preparation of NiCuFe_2_O_4_@mSiO_2_” section and shown in Fig. 1.

**Figure 2:**
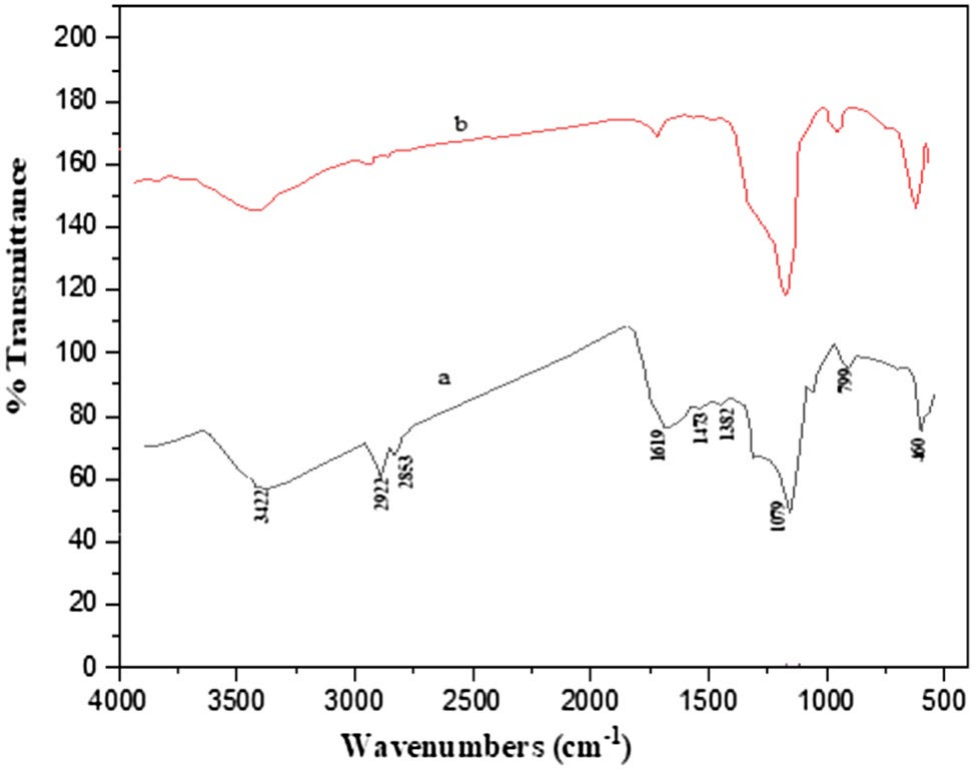
FT-IR spectra of the multi-yolk@shell NiCuFe_2_O_4_ spheres, (**a**) before, and (**b**) after calcination.

The prepared catalyst was characterized by FT-IR, XRD, FE-SEM, EDX, HR-TEM, VSM and BET techniques. FT-IR spectra of the created samples in the region of 400 to 4000 cm^−1^ are shown in Fig. 2. This Figure shows the FT-IR spectra of hollow catalyst before and after the calcination. Figure 2a is shown the spectrum of NiCuFe_2_O_4_@C@SiO_2_ catalyst that was appropriated before calcination. The peaks of 3422 and 1619 cm^−1^ are shown the stretching vibration of the O–H bond and H–O–H bending vibration. The peaks of the 2922, and 2853 cm^−1^ can be appropriated to the stretching vibration of the C–H bond. The peaks of 1473 and 1382 cm^−1^ identify the bending vibration of the C–H bond and the stretching vibration of the C–O bond which shown the template carbon is formed and the CTAB is in the NiCuFe_2_O_4_@C@SiO_2_ catalyst. The peaks that appeared in 460 and 794 cm^−1^ can be related to the Ni–O, Cu–O, and Fe–O bonds. The stretching vibration of the Si–O bond is appeared in 1079 cm^-1^. Figure 2b is shown the spectrum of NiCuFe_2_O_4_@C@SiO_2_ catalyst after calcination. In this step, the absence of the peaks at 2922, 2853, 1473 and 1382 cm^−1^ indicated that the calcination is completely done, the catalyst voided and the NiCuFe_2_O_4_@mSiO_2_ is formed regularly.

**Figure 3:**
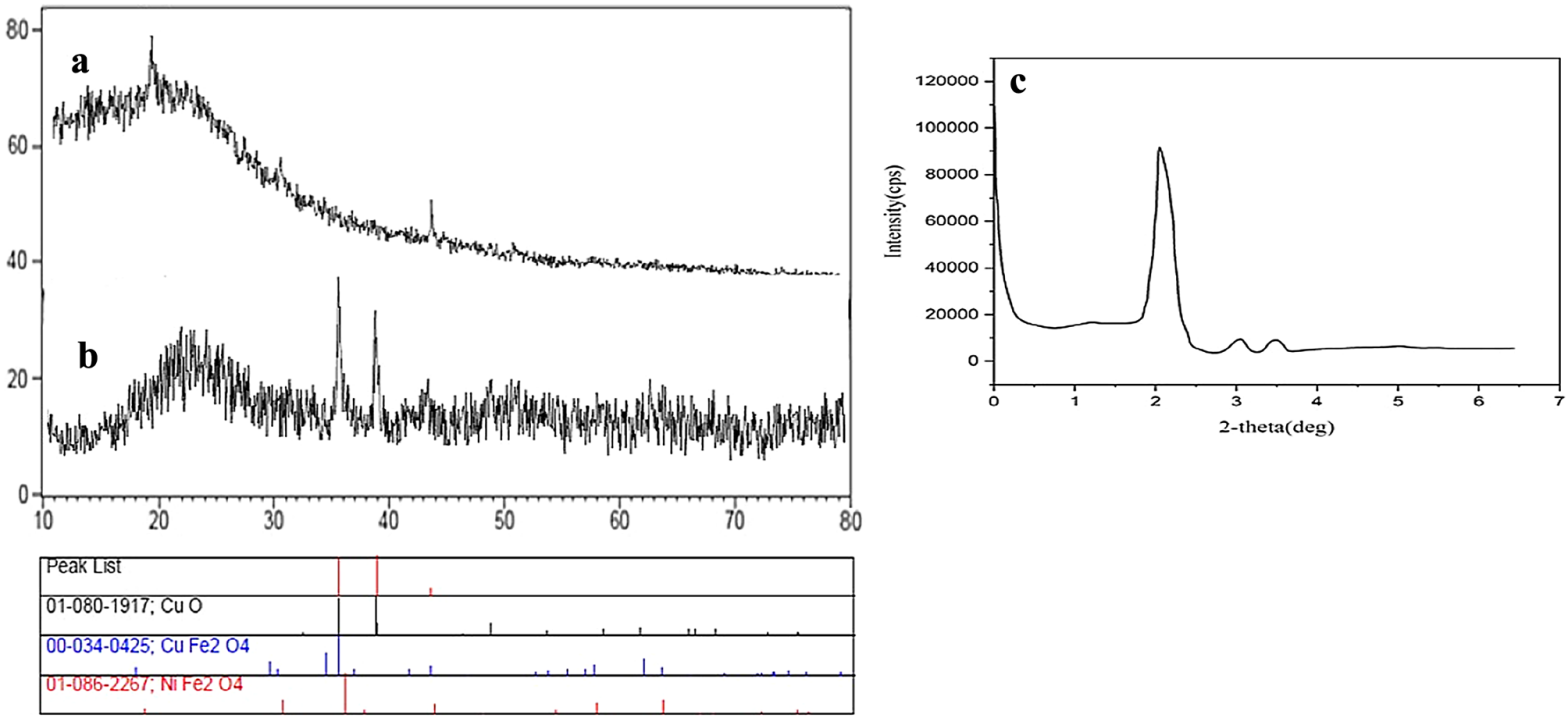
XRD pattern of the NiCuFe_2_O_4_@mSiO_2_ spheres (**a**) before, (**b**) after calcination and (**c**) XRD low angle of the NiCuFe_2_O_4_@mSiO_2_ spheres.

The XRD pattern of multi-yolk@shell NiCuFe_2_O_4_ is shown in Fig. 3 which the related peaks are shown. Figure 3a shows the catalyst before calcination that it is irregularly indicated. The presence of the peaks at 2Ɵ = 20.2, 33, and 36.6 in Fig. 3b can be appropriated to the catalyst before calcination that shows the catalyst is regularly made. Figure 3c shows the XRD low angle of catalyst and the presence of the sharp peak at 2Ɵ indicates that multi-yolk@shell NiCuFe_2_O_4_ mesoporous silica catalyst is made.

**Figure 4:**
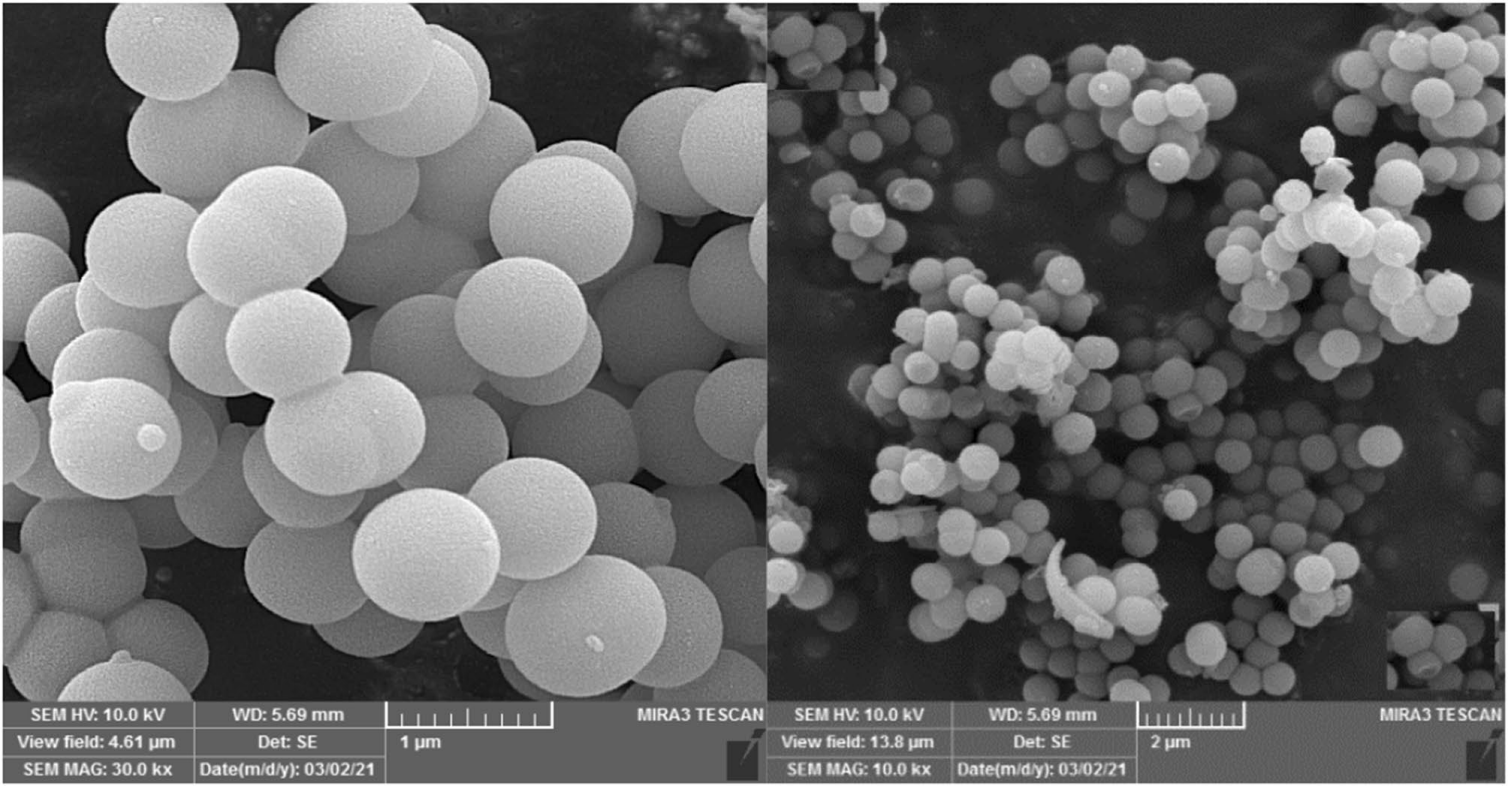
FE-SEM images of the multi-yolk@shell NiCuFe_2_O_4_@mSiO_2_ nanospheres.

Figure 4 shows the field emission scanning electron microscopy (FE-SEM) images for hollow multi-shell NiCuFe_2_O_4_@mSiO_2_ spheres catalyst. The FE-SEM images (Fig. 4) is indicated that the catalyst is made up of uniform, spheres, and hollow.

**Figure 5:**
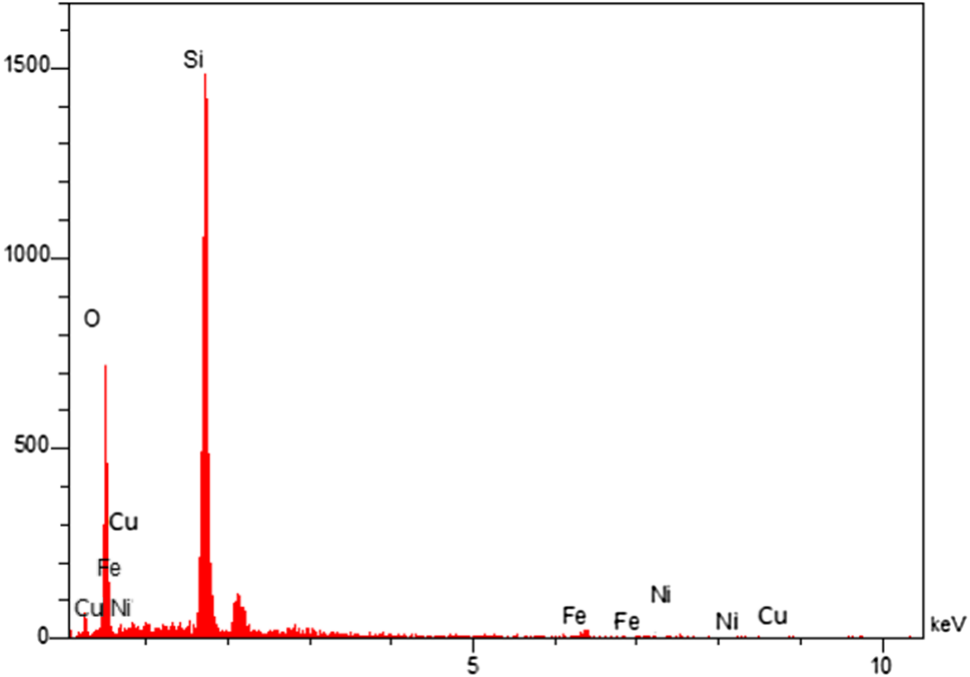
EDX spectrum of the multi-yolk@shell NiCuFe_2_O_4_@mSiO_2_ spheres.

Furthermore, Fig. 5 is demonstrated the EDX analysis of the hollow multi shell NiCuFe_2_O_4_@mSiO_2_ spheres. This result is indicated that the nanospheres included the Ni, Cu, O, Si, and Fe elements. This finding is quite following the FT-IR results.

**Figure 6:**
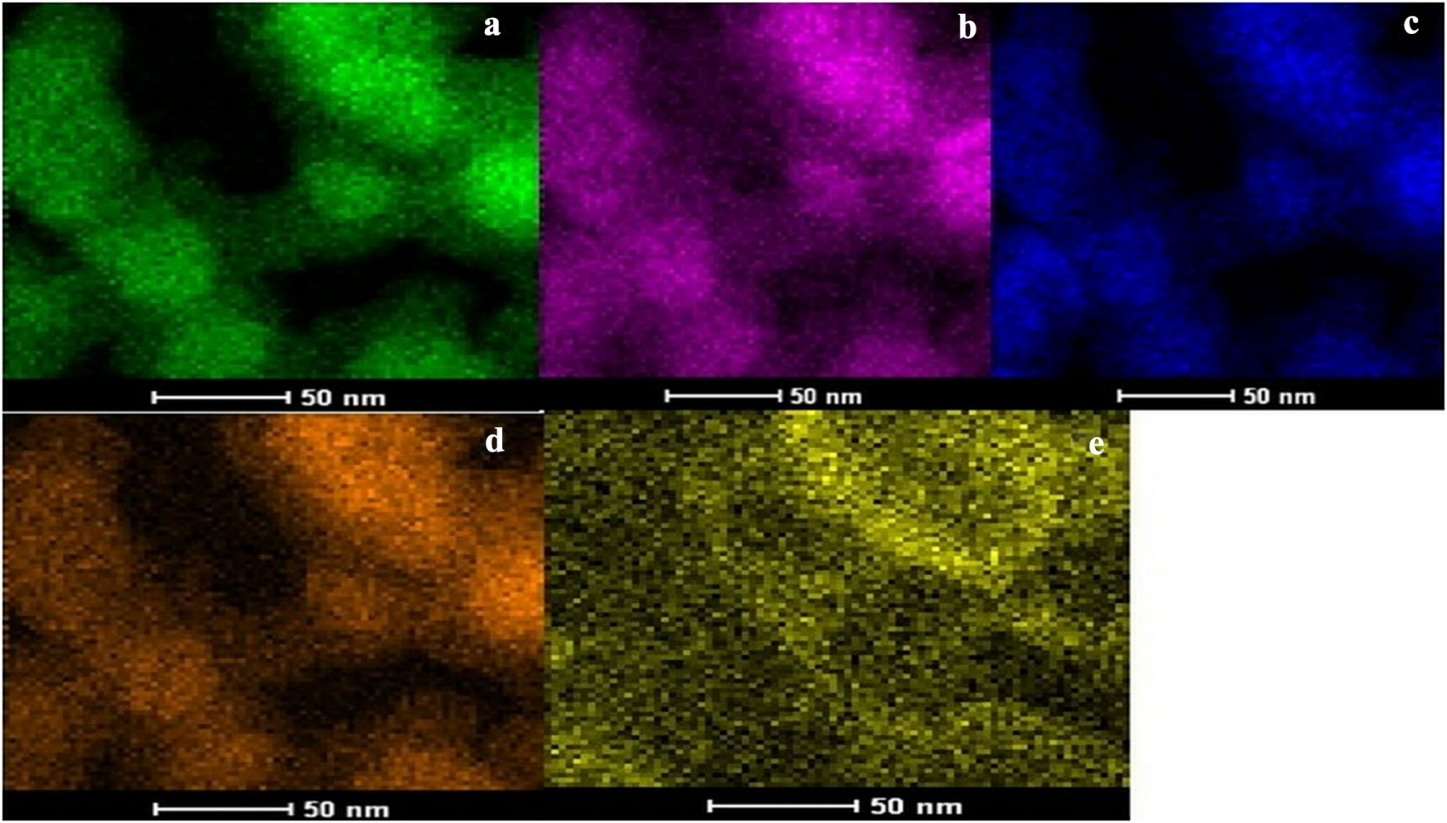
Elemental mapping analysis: for (**a**) Fe, (**b**) Cu, (**c**) Ni, (**d**) O, (**e**) Si.

Also, the results of elemental mapping analysis is indicated the Fe, Cu, Ni, O, and Si elements (Fig. 6). This result is quite following the EDX analysis.

**Figure 7:**
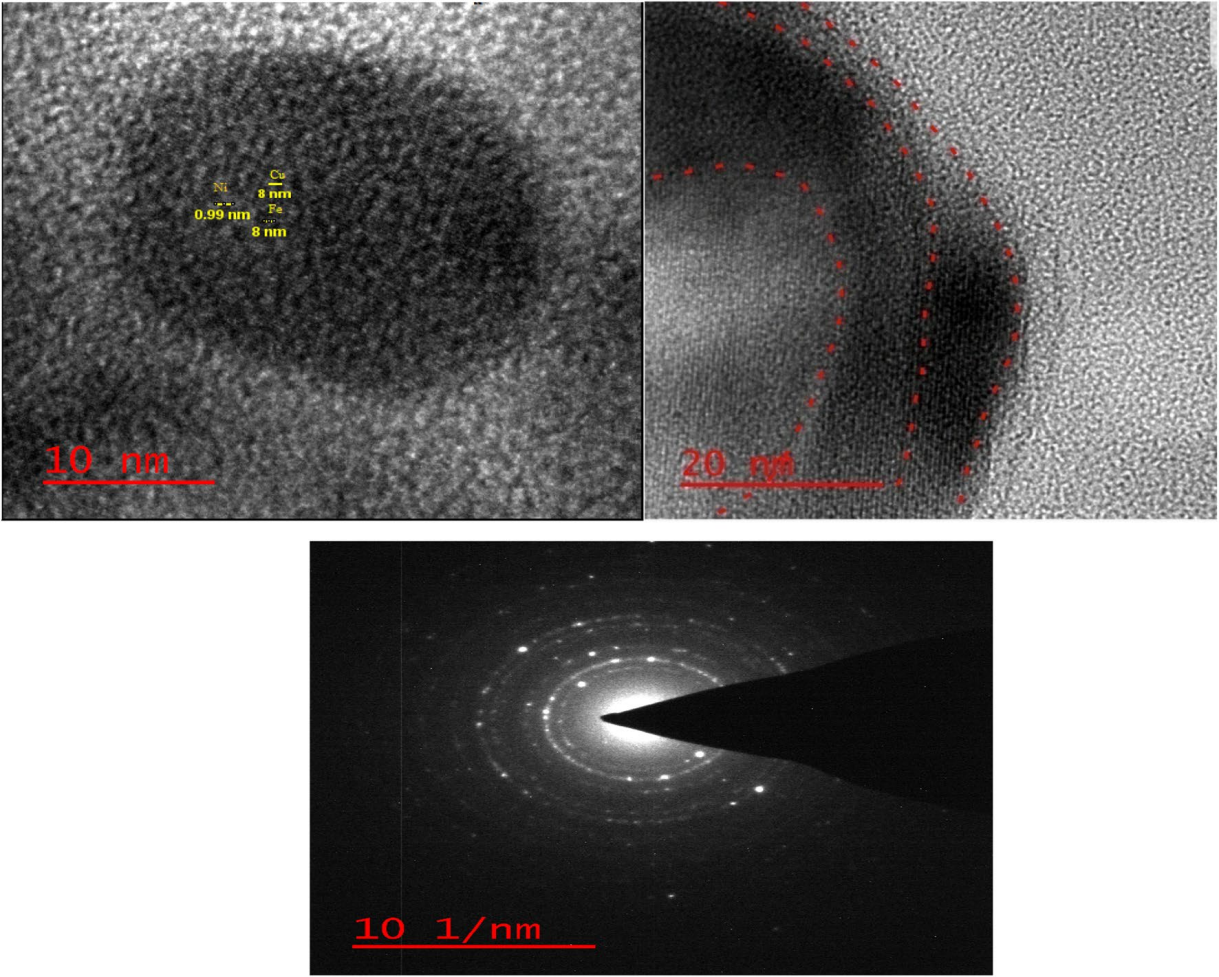
High resolution TEM (HR-TEM) of the multi-yolk@shell NiCuFe_2_O_4_@mSiO_2_.

The high resolution TEM (HR-TEM) of the multi-yolk@shell NiCuFe_2_O_4_@mSiO_2_ spheres indicates the structure of the catalyst in the Fig. 7. These images show that the catalyst is made in a sphere uniform, multi-shell, yolk–shell and is multi-metal.

**Figure 8:**
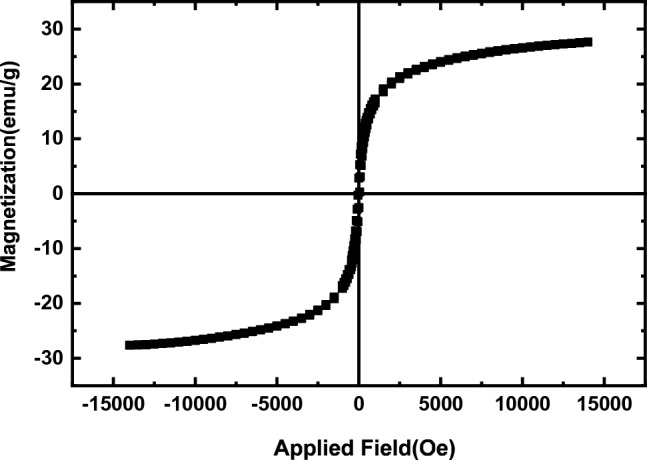
VSM analysis of the multi-yolk@shell NiCuFe_2_O_4_@mSiO_2_ spheres.

A vibrating sample magnetometer (VSM) has been used to explore the magnetic properties of various materials for NiCuFe_2_O_4_@mSiO_2_ spheres magnetization pattern are shown in Fig. 8. The multi-yolk@shell NiCuFe_2_O_4_@mSiO_2_ spheres have a 28.1 emu per g magnetic valence.

**Figure 9:**
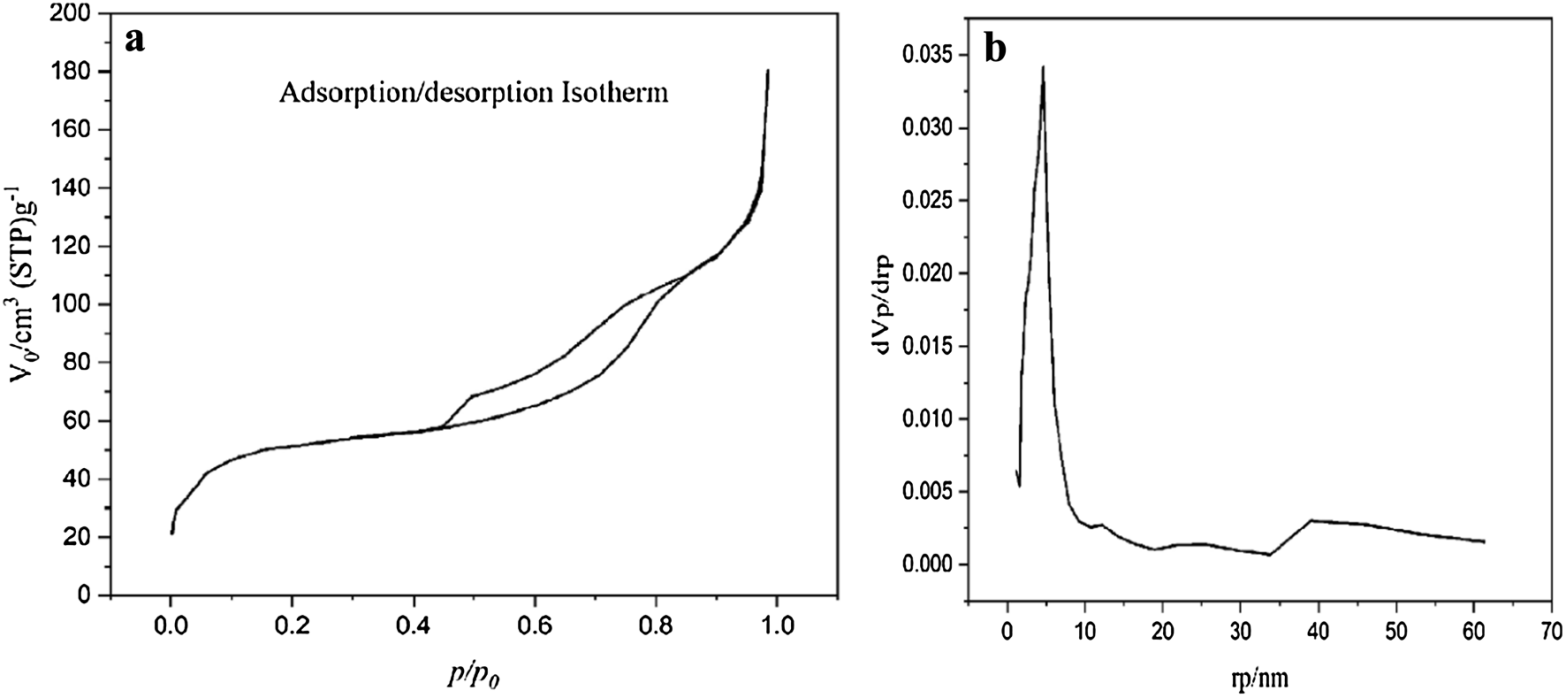
The nitrogen adsorption–desorption isotherm (**a**) and corresponding BJH pore size (**b**) of multi-yolk@shell NiCuF_2_O_4_@mSiO_2._

The nitrogen adsorption–desorption isotherm of the hollow multi-shell NiCuFe_2_O_4_@mSiO_2_ and corresponding BJH pore size distribution is shown in Fig. 9. The BJH is shown an average pore size of 4.6 nm which indicated the catalyst is mesoporous (Fig. 9b). Due to the mesoporous silica, the available surface area is 180.51 m^2^/g that higher than the catalyst reported without mesoporous silica (Fig. 9a)^42^.

### Investigation of catalytic activity

In order to assess its ability as just a catalyst for the synthesis of tetrahydronaphtalene, multi-yolk@shell NiCuF_2_O_4_@mSiO_2_ was investigated (Table 1). The reaction of cyclohexanone, aromatic aldehyde and the 4-Br-benzaldehyde was selected as a model reaction. So, some studies of the influence of heat, suitability of solvent, and nanocatalyst amounts loading on the reaction speed was conducted (Table 1). This Table shows the low product yields when using the ethanol, water, ethanol–water and, methanol as solvent (Table 1, entries 1–4). In this research, the best conditions for the model reaction at 50 °C to create the tetrahydronaphtalene derivative are reported to be solvent-free (Table 1, entry 5).

**Table 1:** Optimization of the reaction conditions.


Entry	Solvent	T (°C)	Catalyst amount (mg)	Time (min)	Yield (%)^a^
1	Ethanol	Reflux	2	60	93
2	H_2_O	Reflux	2	45	85
3	Ethanol/H_2_O	Reflux	2	50	90
4	Methanol	Reflux	3	65	90
5	Solvent free	50	1	60	98

Reaction conditions: 4-Br-benzaldehyde (1 mmol), cyclohexanone (1 mmol), malononitril (2 mmol).

^a^Isolated yield.

Several kinds of catalysts for this reaction were examined once the reaction parameters were optimized (Table 2). When the present catalyst was compared with the other catalysts, such as; NiFe_2_O_4_, HCl and H_2_SO_4_, it was found that the reaction in the presence of multi-yolk@shell NiCuFe_2_O_4_@mSiO_2_ spheres as a catalyst, obtained the highest product yield. As a result, the multi-yolk@shell NiCuFe_2_O_4_@mSiO_2_ spheres are preferable to other catalysts because of short reaction time, low catalyst loading and highest product yield.

**Table 2:** Catalytic activity of the different catalysts for synthesis of tetrahydronaphtalene*.*

Entry	Catalyst	Time	Yield%
1	No catalyst	280	–
2	NiFe_2_O_4_	210	90
3	HCl	210	86
4	H_2_SO_4_	180	86
5	Multi-yolk@shell NiCuFe_2_O_4_@mSiO_2_	60	98

Following the adjustment of the reaction conditions including temperature, catalyst loading, and different solvents, the development and aim of our methodology for the production of various tetrahydronaphtalenes were carried out. This study looked at how different benzaldehydes reacted with cyclohexanone derivatives, malononitrile, and the corresponding results are summarized in Table 3. As can be seen in this Table, the malononitrile effectively reacted with a variety of cyclohexanones and benzaldehydes including electron-withdrawing and electron-donating substituents. The results are shown that the necessary tetrahydronaphtalenes, including different substituents, are synthesized in the majority of cases in high yields and short reaction times. So that the present reaction was absolutely uniform.

**Table 3:** Synthesis of tetrahydronaphtalene derivatives.


Entry	R_1_	R_2_	Product	Time (min)	Yield^a^ (%)	m.p (°C)
1	H	H		70	97	255–258
2	4-Br	H		60	98	243–245
3	4-NO_2_	H		60	98	263–266
4	4-N(CH_3_)_2_	H		65	96	264–265
5	4-F	H		60	98	263–267
6	4-Cl	H		65	95	246–249
7	4-OCH_3_	H		70	93	258–261
8	4-CH_3_	H		70	92	234–236
9	3-Br	H		75	94	248–253
10	H	CH_3_		70	96	260–262
11	4-Br	CH_3_		65	97	253–257
12	4-OH	CH_3_		65	96	256–257
13	4-Cl	CH_3_		65	97	236–240

Reaction conditions: derivatives benzaldehyde (1 mmol), cyclohexanone derivatives (1 mmol), malononitrile (2 mmol) and multi-yolk@shell NiCuFe_2_O_4_@mSiO_2_ (1 mg).

^a^Isolated yield.

Furthermore, the comparison of tetrahydronaphtalenes synthesis using the present nanocatalyst with the previously reported catalysts are reported in Table 4. As indicated in this Table, the works using different catalysts have disadvantages than the using multi-yolk@shell NiCuFe_2_O_4_@mSiO_2_ catalyst based on yields, reaction times and catalyst loading amounts (Table 4, entries 1–5 *vs* 6).

**Table 4:** Comparison of the present catalyst with the previously reported catalysts.

Entry	Catalyst	Conditions	Time (min)	Yield (%)	Ref.
1	[BPy]BF_4_	Solvent free/60 °C	300	83	^41^
2	NH_4_OAc	EtOH-H_2_O/50–60 °C	120	90	^43^
3	Ethylene diamine	MeOH/r.t	1440	86	^44^
4	OBS	EtOH/reflux	360	93	^29^
5	Nanostructured diphosphate Na_2_CaP_2_O_7_	EtOH/reflux	240	92	^45^
6	multi-yolk@shell NiCuFe_2_O_4_@mSiO_2_	Solvent free/50 °C	60	98	This work

### Proposed reaction mechanism

**Scheme 1:**
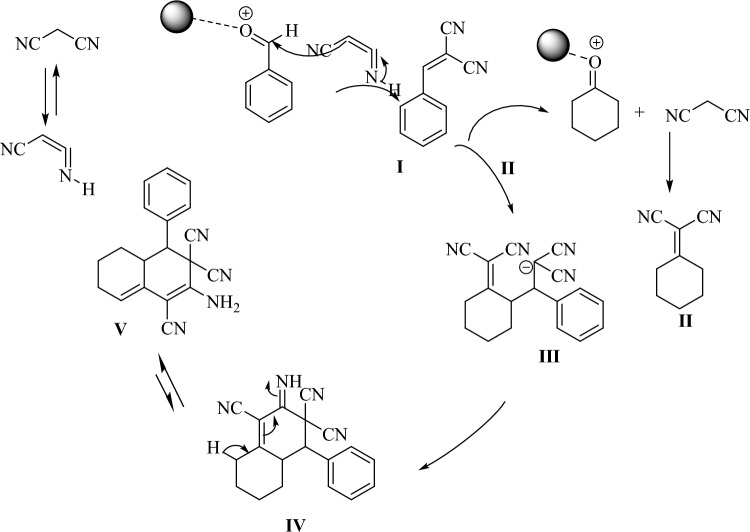
The proposed reaction mechanism for the formation of tetrahydronaphtalene.

Scheme 1 shows a plausible reaction mechanism for the formation of tetrahydronaphtalen. The mesoporous silica can be lead to highly large surface area in the catalyst and coordinating agent with the carbonyl group of aldehyde to give the product in higher isolated yield. In this reaction, at the first time, the catalyst as a Lewis acid activated the benzaldehyde, then the activated benzaldehyde reacted with malononitrile and created an intermediate **I**. Then, the cyclohexanone reacted with another malononitrile molecule, an intermediate **II** is produced. Subsequently, the reaction between **I** and **II** carried out to obtain the intermediate **III**. The cyclization reaction of **III** through intramolecular nucleophilic addition of carbanion into cyano group to form **IV**. Finally, the isomerization of **IV** to give **V** compound as a target product.

**Figure 10:**
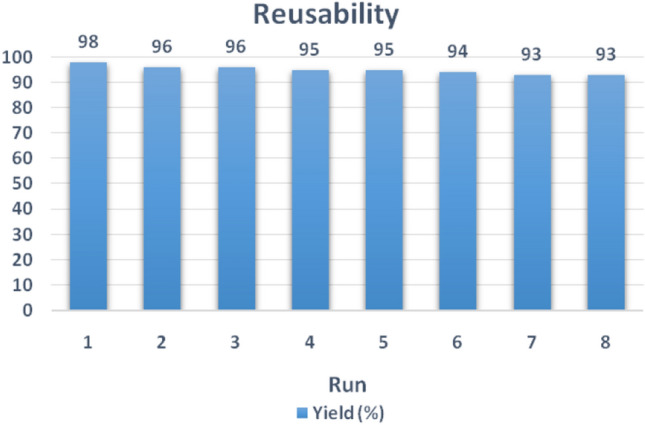
Reusability of the multi-yolk@shell NiCuFe_2_O_4_@mSiO_2_ spheres after 8 times used in the reaction.

In order to study the reusability of catalyst, after completion of the reaction, the catalyst is separated by an external magnet, then, washed with ethanol and dried. The benzaldehyde with cyclohexanone derivative and malononitrile was chosen and reacted together as a model reaction to measure the catalyst reusability. Then, Fig. 10 is shown the multi-yolk@shell NiCuFe_2_O_4_@mSiO_2_ nanospheres can be reused in the reaction after 8 times without loss activity.

## Conclusion

In this research, the multi-yolk@shell NiCuFe_2_O_4_@mSiO_2_ spheres was designed and prepared. Then, it was used as a reactive nanocatalyst for synthesis of tetrahydronaphtalene derivatives under solvent free conditions. The target products were purely obtained in highly yields and short reaction times. The XRD and EDX are indicated the existence of metals in the catalyst. HR-TEM of the catalyst is shown the confirmation of multi-yolk@shell and multi metallic structure. It was found that according to VSM analysis of catalyst, it has high magnetic property. Therefore, the catalyst was easily separated from the reaction by an external magnet and reused in the reaction 8 times without any decrease the catalytic reactivity.

### Supplementary Information

Supplementary Information.

## Data Availability

All data generated or analysed during this study are included in this published article [and its Supplementary Information files].
