# Supplementary material for: Design, fabrication and characterization of mesoporous yolk–shell nanocomposites as a sustainable heterogeneous nanocatalyst for synthesis of ortho-aminocarbonitrile tetrahydronaphthalenes
Source: Sci Rep. 2023 Dec 18;13:22464. doi: 10.1038/s41598-023-50021-7 (PMC10725875; doi:10.1038/s41598-023-50021-7)
Supplement: Supplementary file 1 — Supplementary Information. [file 41598_2023_50021_MOESM1_ESM.pdf]

Supporting information

Design, fabrication and characterization of mesoporous yolk-shell nanocomposites as a sustainable heterogeneous nanocatalyst for synthesis of ortho-aminocarbonitrile tetrahydronaphthalenes

Somayeh Kazempour, HosseinNaeimi*

Department of Organic Chemistry, Faculty of Chemistry, University of Kashan, Kashan, 87317-51167, I.R. Iran; Tel: 98-31-55912388; Fax: 983155912397; E-mail: Naeimi@kashanu.ac.ir

A typical procedure for the tetrahydronaphthalene

Tetrahydronaphthalenesynthesized from the multi-component reaction. In this process, cyclohexanon derivatives (1mmol), malononitrile (2mmol), aromatic aldehyde (1mmol), and 1mg $NiCuFe_2O_4@mSiO_2$as catalyst are mixed under stirring at 50 ^{0}C. The progress of the reaction was monitored by thin-layer chromatography (TLC). After completion of the reaction, ethanol (3 mL) was added and the catalyst was separated by an external magnet fromthe reaction mixtureand washed with ethanol (3mL). Then, the crude product was filtered off and recrystallized from ethanol to obtain the pure products.

The FT-IR and ^{1}H NMR spectra of products:

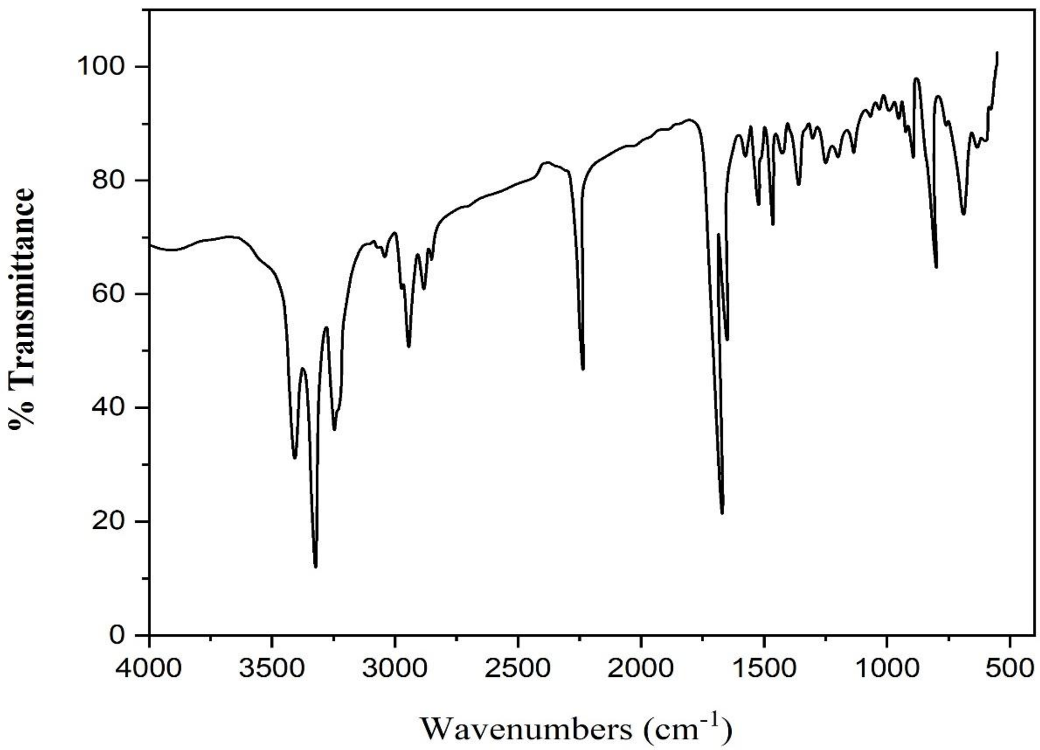

IR of 4a

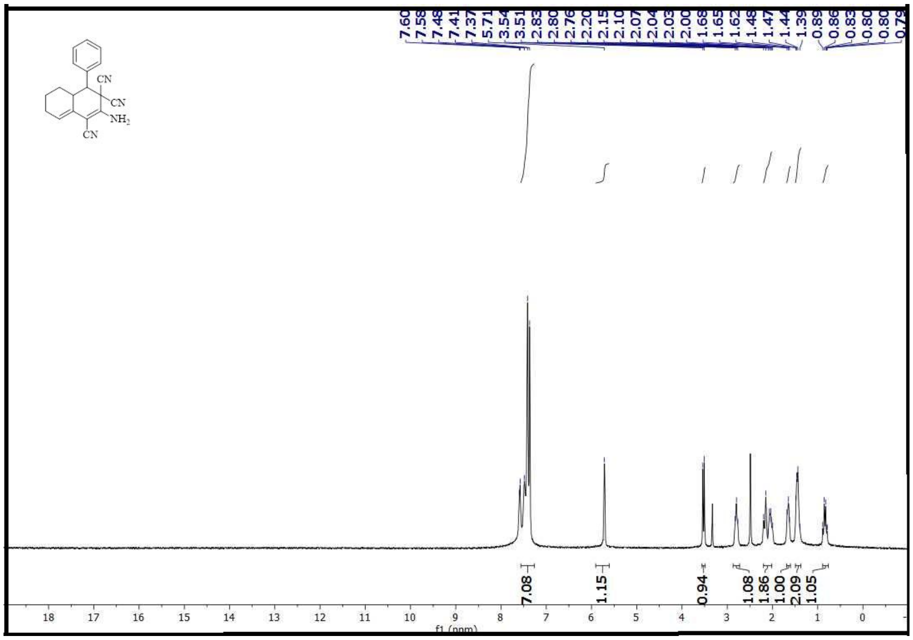

^{1}H NMR of 4a

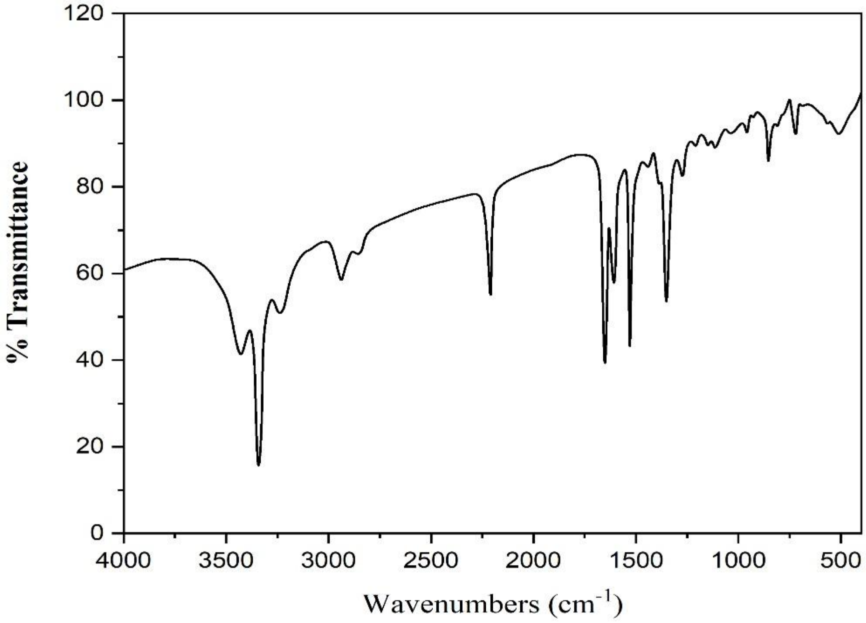

IR of 4b

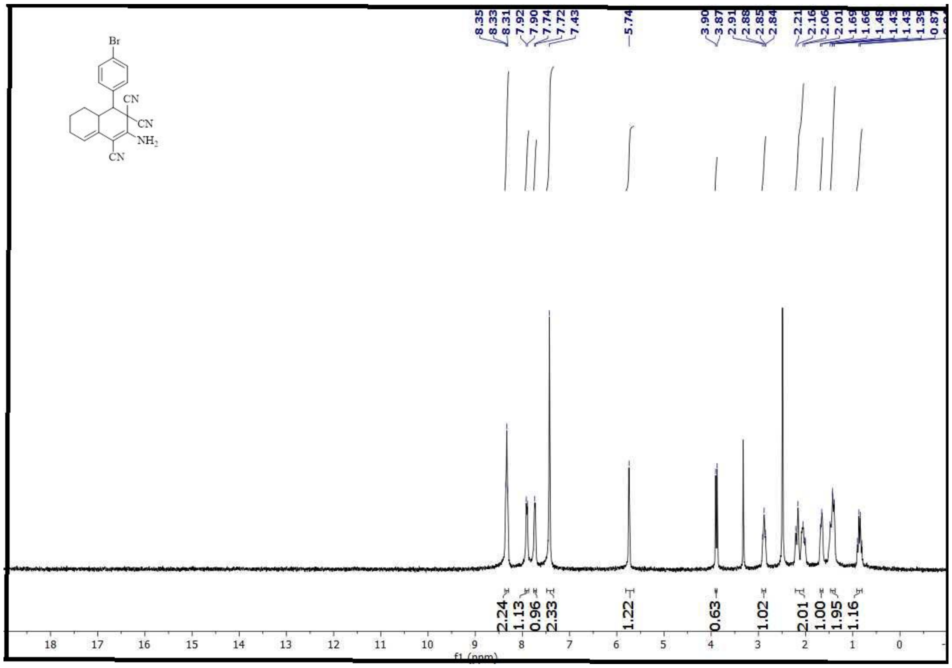

^{1}H NMR of 4b

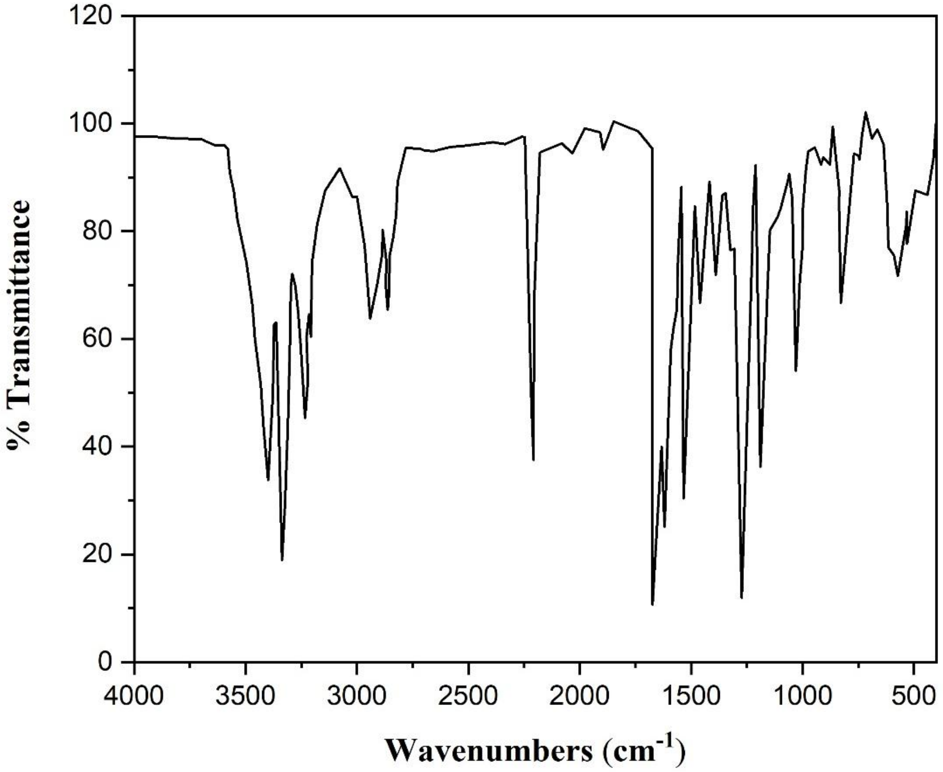

IR of 4c

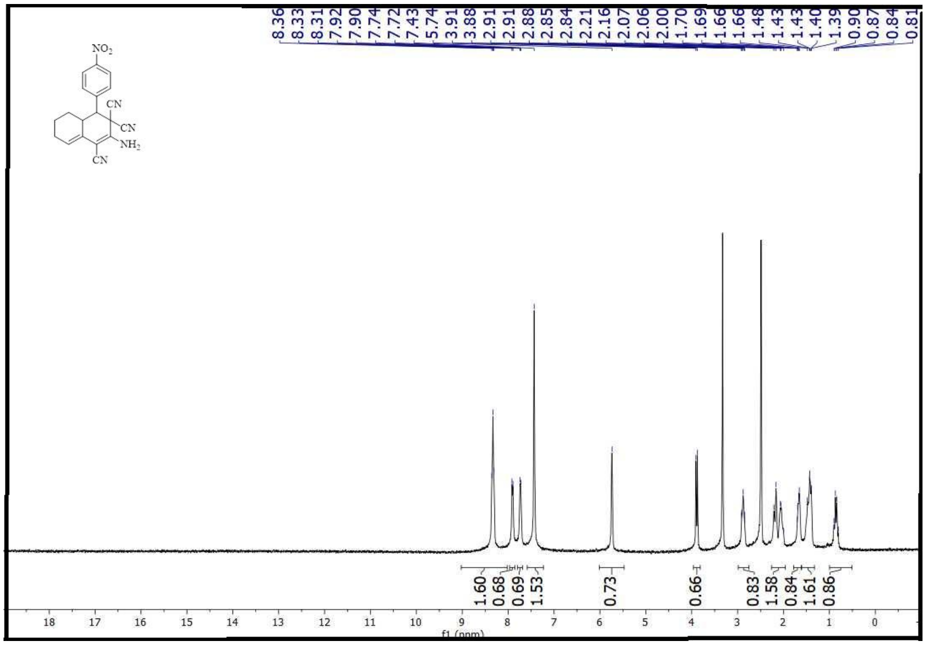

^{1}H NMR of 4c

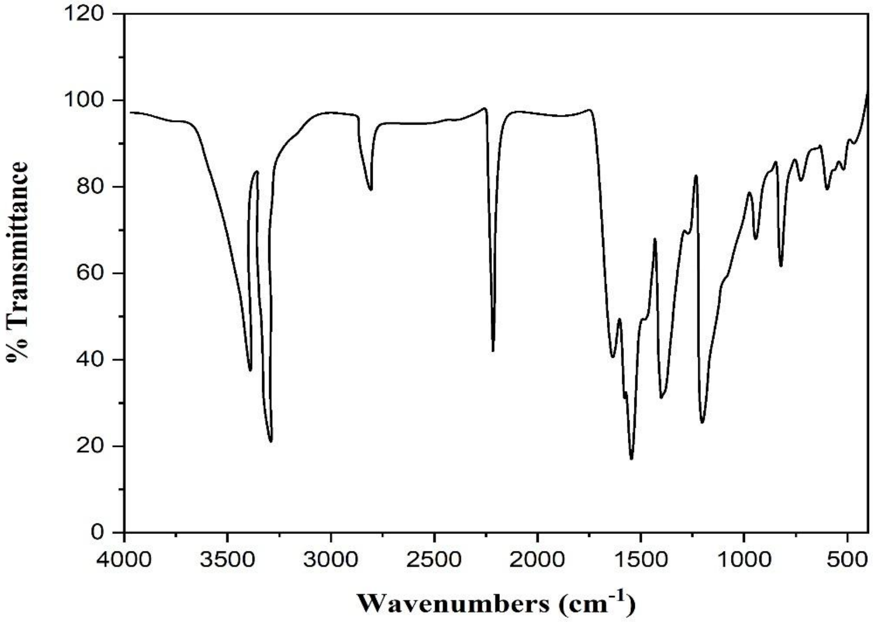

IR of 4d

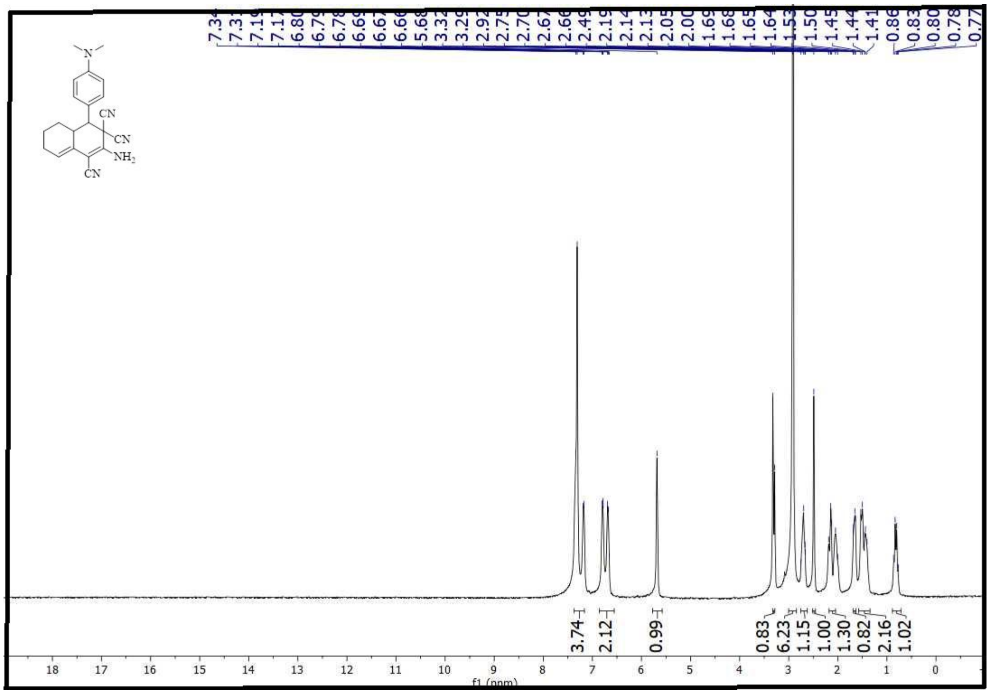

^1H NMR of 4d

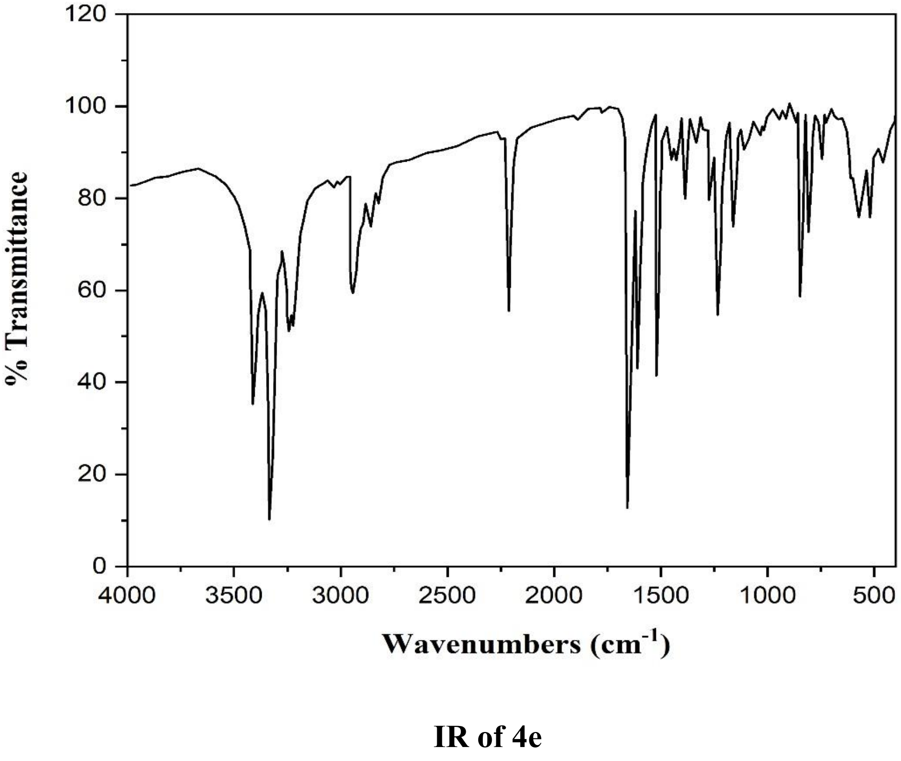

IR of 4e

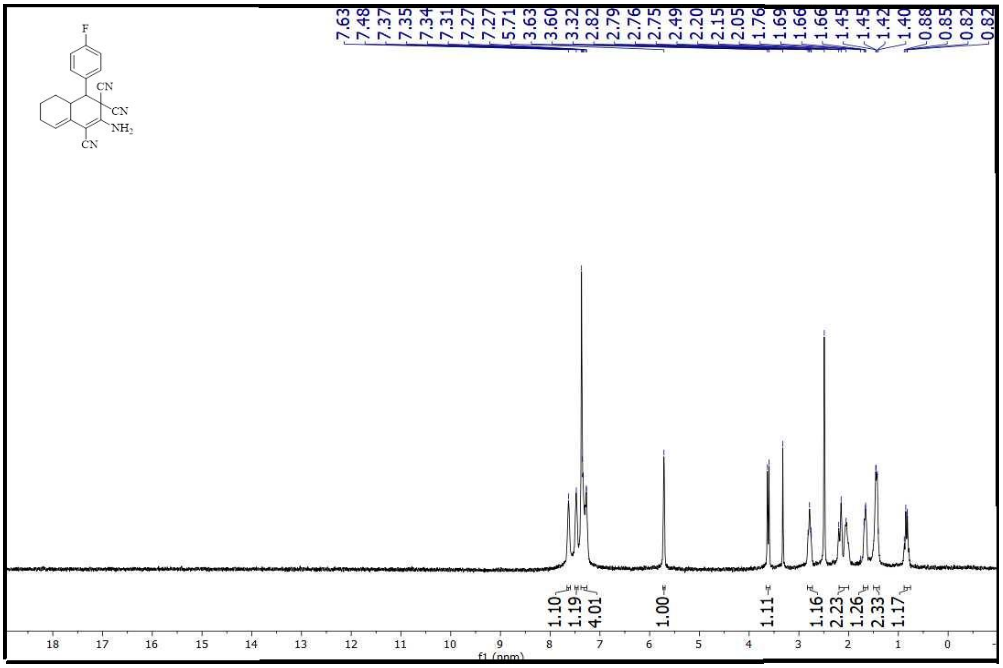

^{1}H NMR of 4e

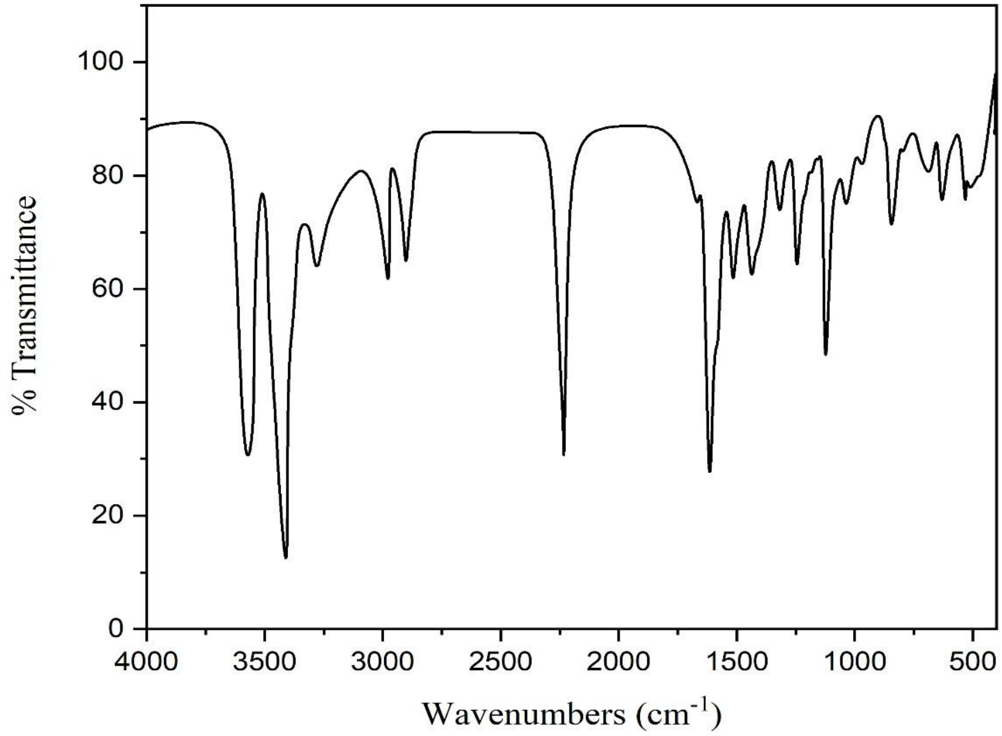

IR of 4f

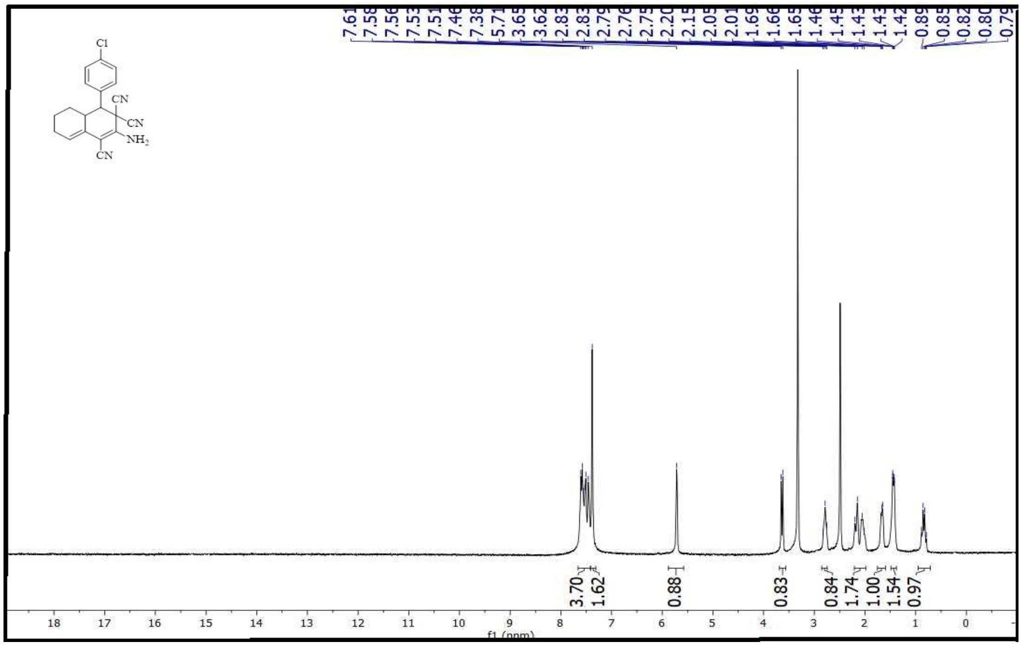

^{1}H NMR of 4f

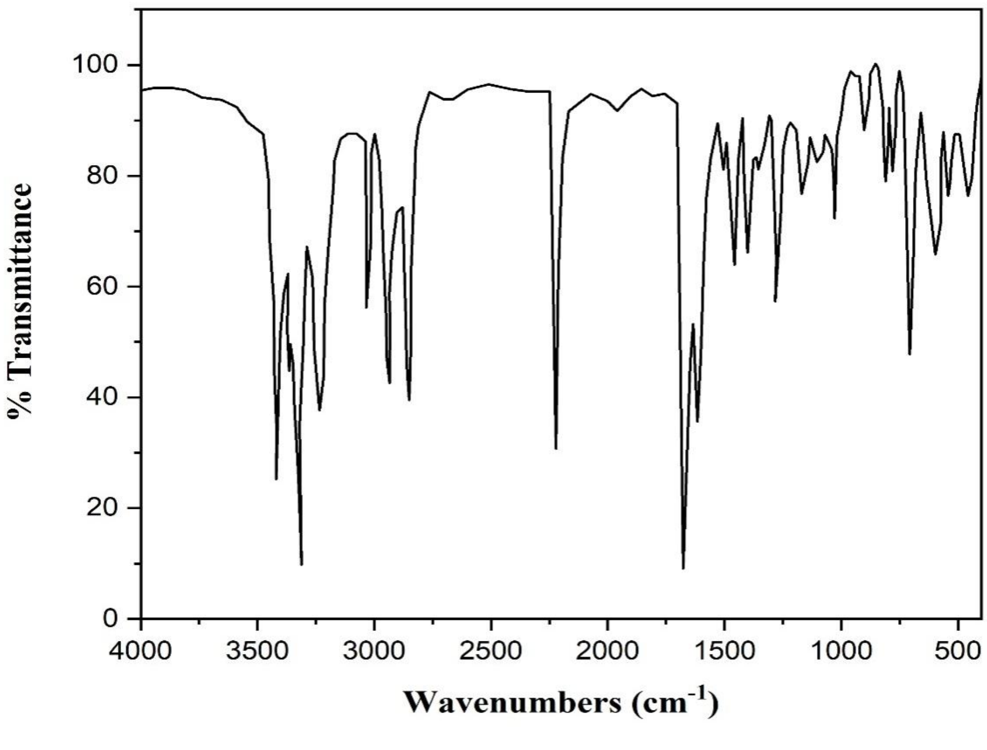

IR of 4g

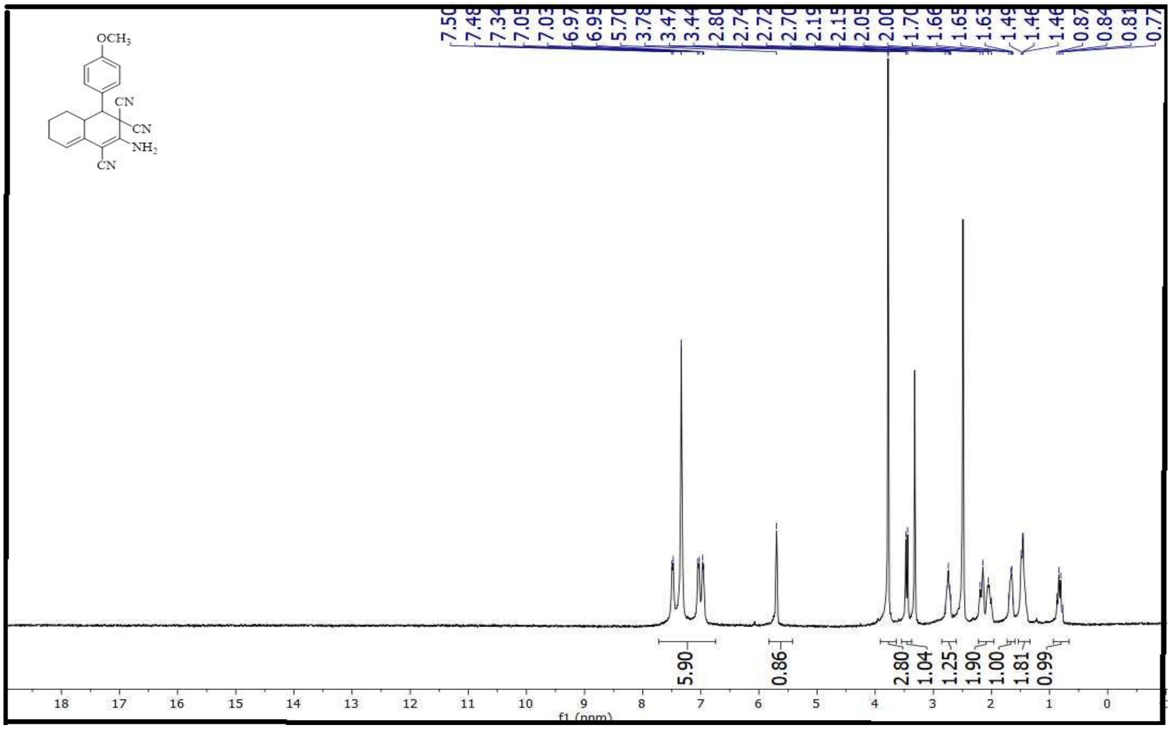

^{1}H NMR of 4g

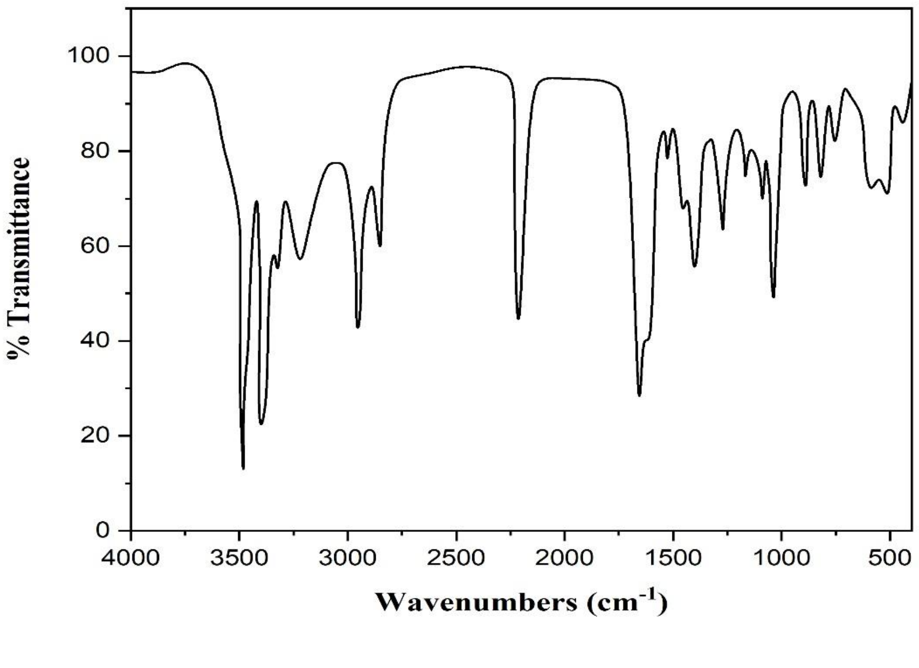

IR of 4h

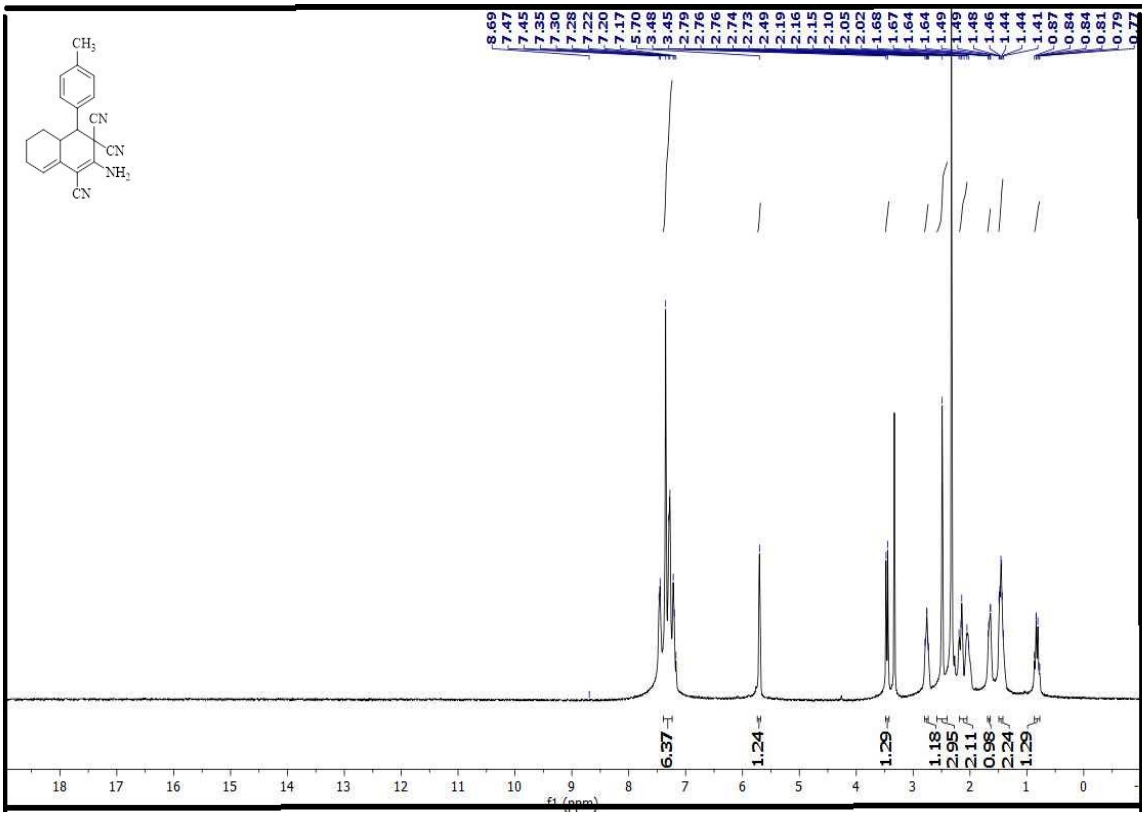

^{1}H NMR of 4h

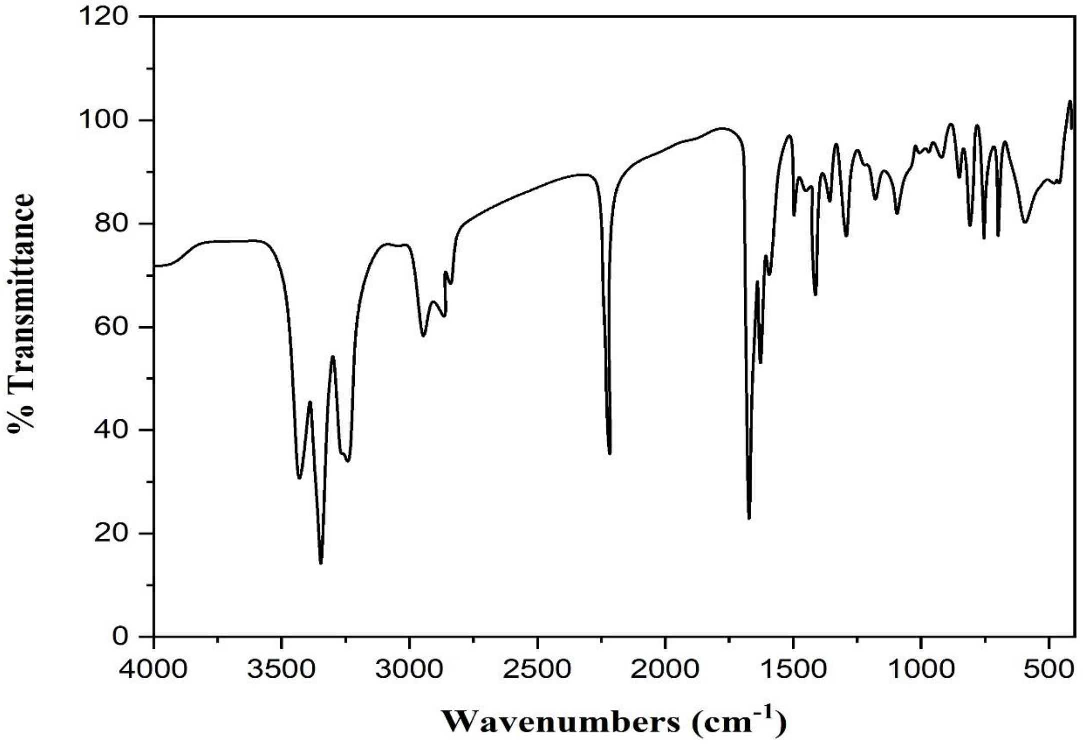

IR of 4i

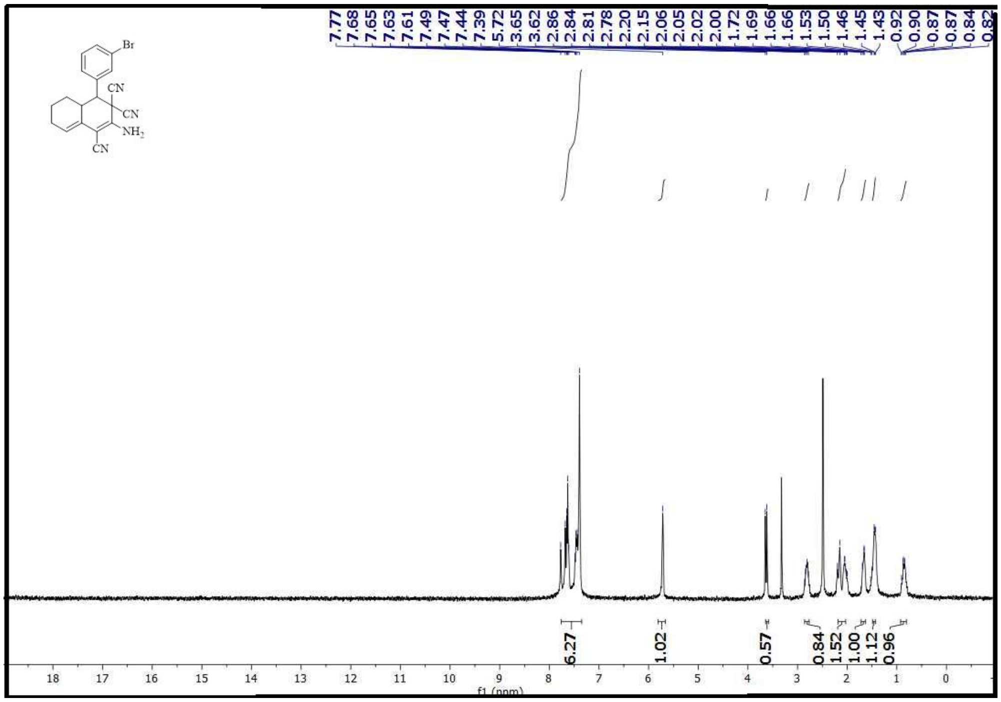

H NMR of 4i

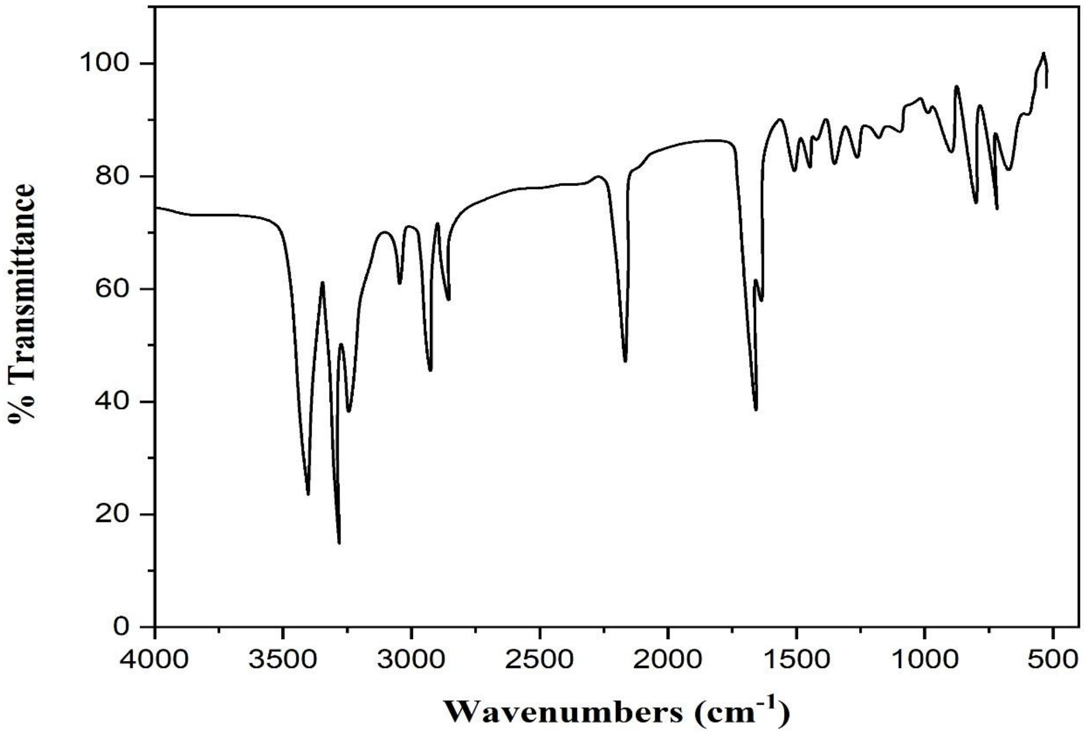

IR of 4j

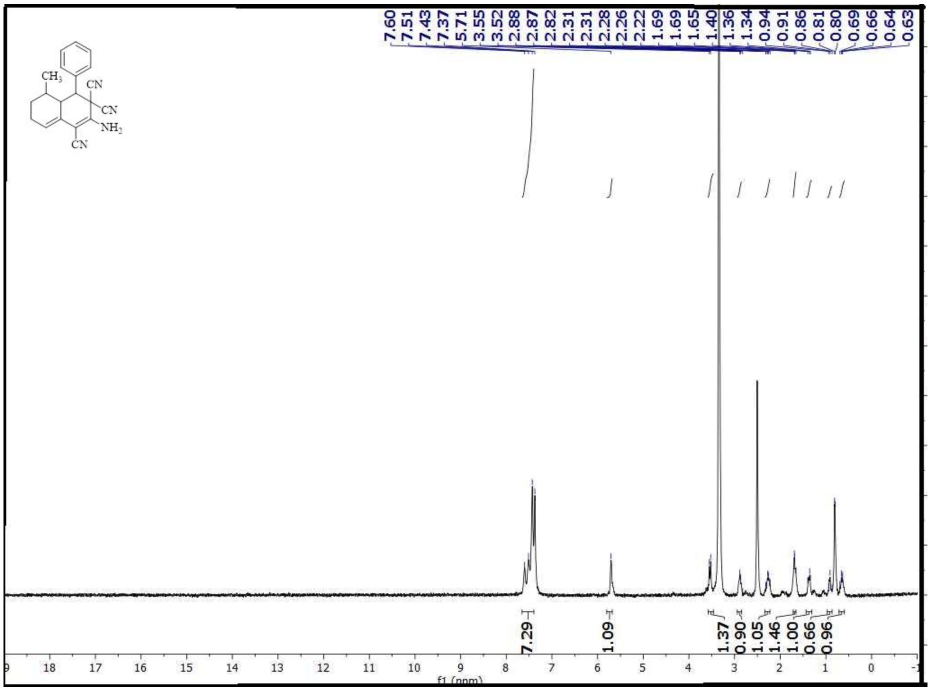

^{1}H NMR of 4j

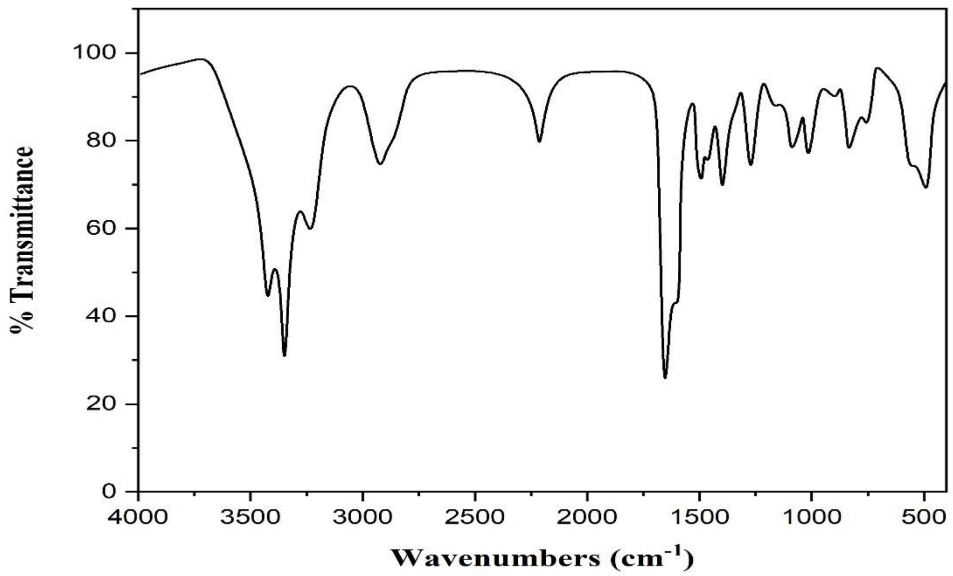

IR of 4k

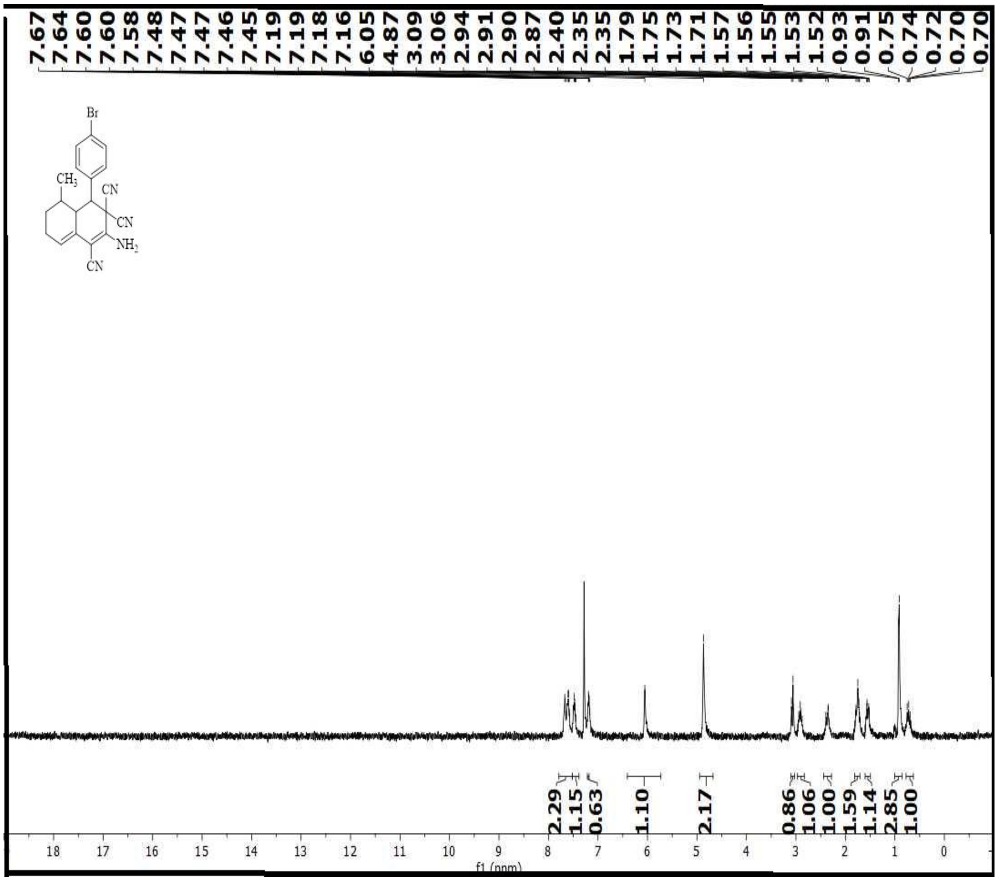

^{1}H NMR of 4k

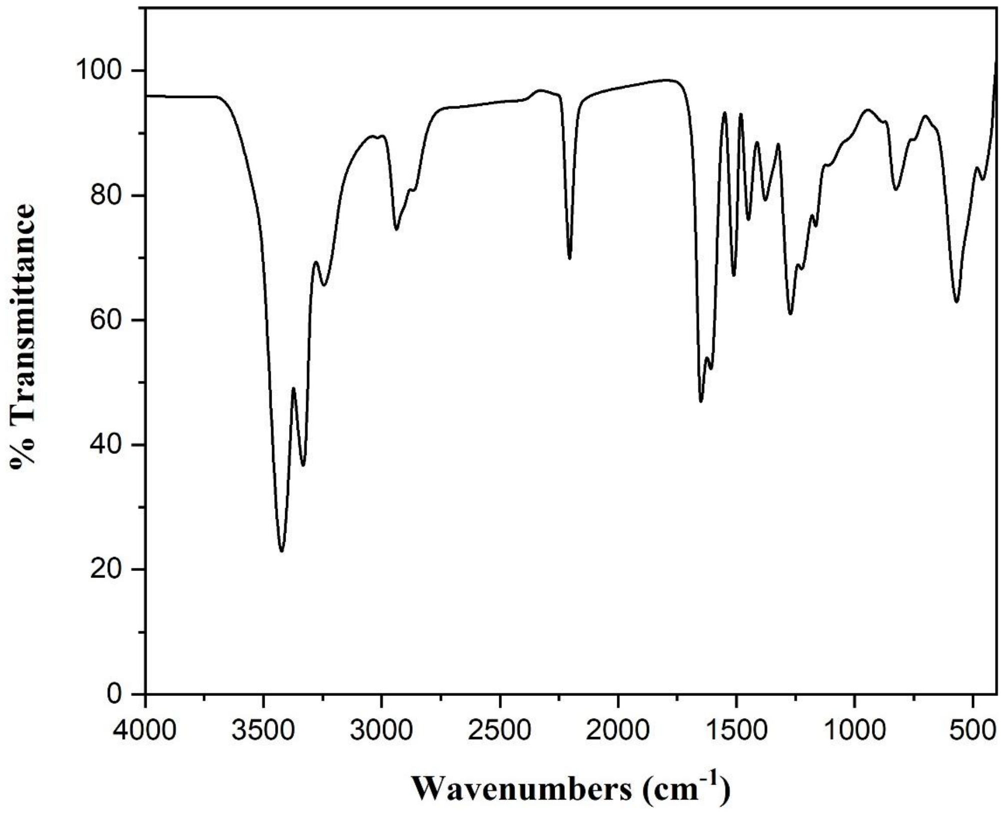

IR of 4l

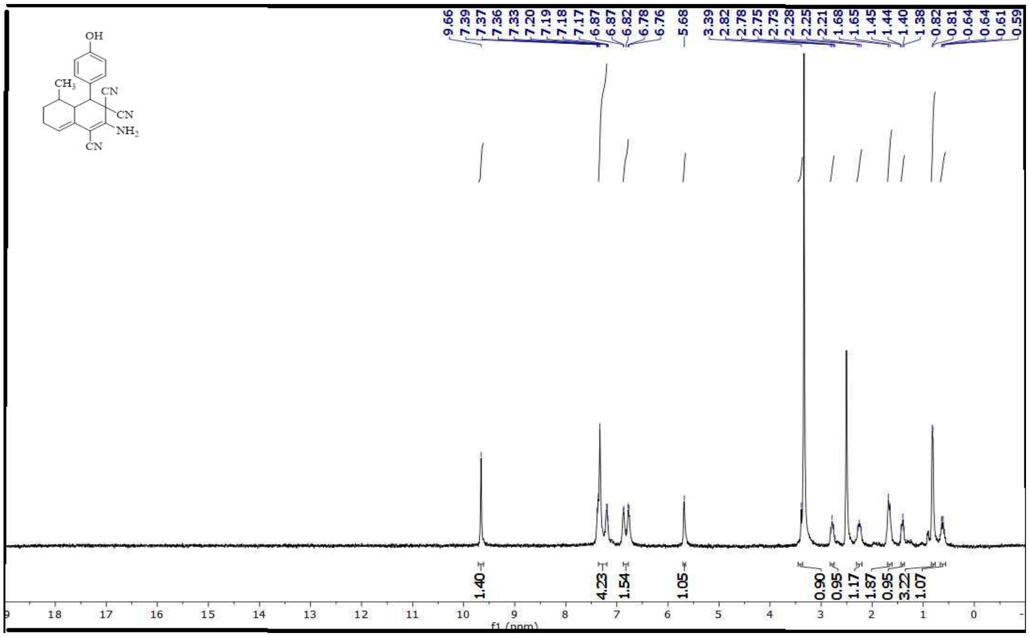

^{1}H NMR of 4l

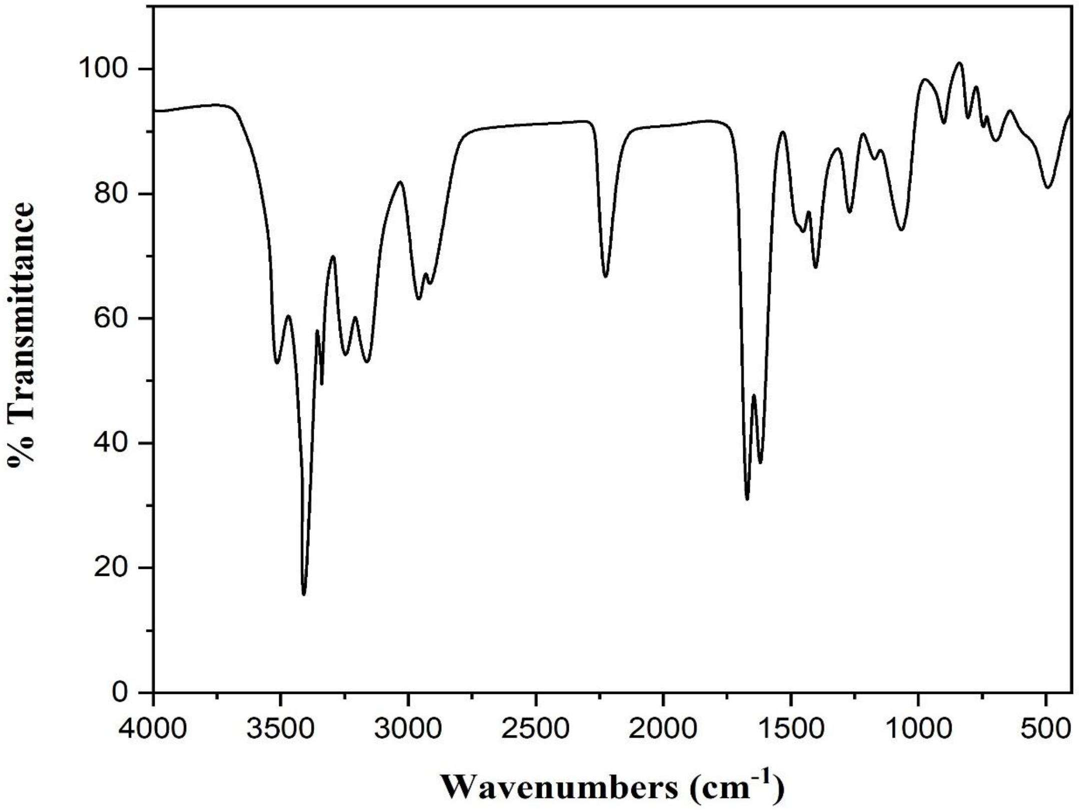

IR of 4m

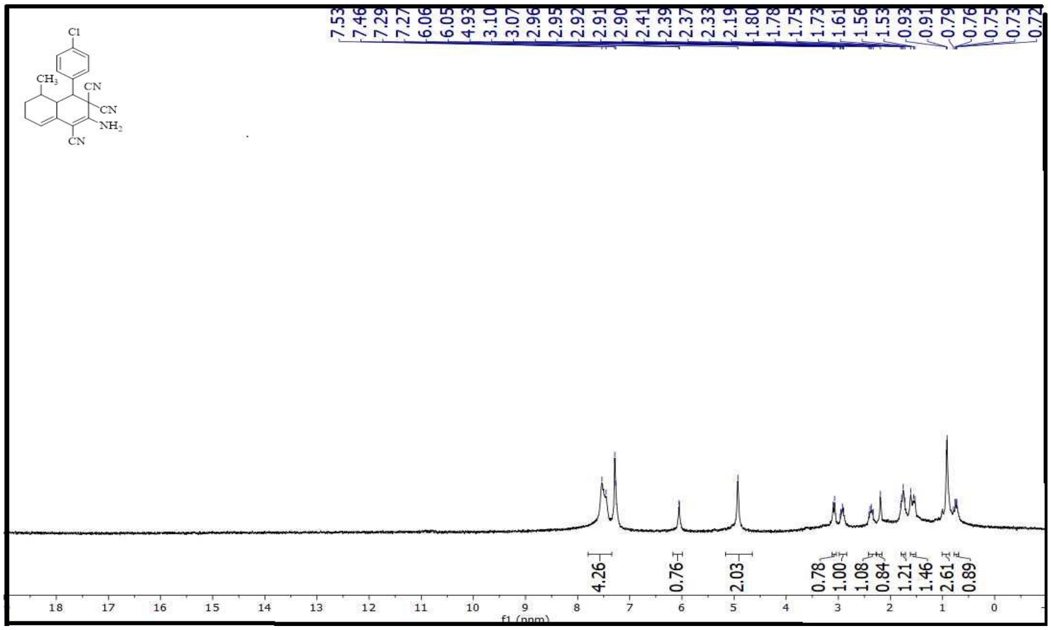

^{1}H NMR of 4m
